# Systematic reviews as a “lens of evidence”: Determinants of participation in breast cancer screening

**DOI:** 10.1177/0969141320930743

**Published:** 2020-06-09

**Authors:** O Mandrik, E Tolma, N Zielonke, F Meheus, C Ordóñez-Reyes, JL Severens, R Murillo

**Affiliations:** 1 School of Health and Related Research, Health Economic and Decision Science (HEDS), The University of Sheffield, Sheffield, UK; 2 Erasmus School of Health Policy & Management, Erasmus University Rotterdam, Rotterdam, The Netherlands; 3 Section of Early Detection and Prevention, International Agency for Research on Cancer, Lyon, France; 4 Faculty of Public Health, Kuwait University, Jabriya, Kuwait; 5 Department of Public Health, Erasmus MC, University Medical Center Rotterdam, Rotterdam, The Netherlands; 6 Centro Javeriano de Oncología, Hospital Universitario San Ignacio, Bogotá, Colombia; 7 Institute for Medical Technology Assessment (iMTA), Erasmus University Rotterdam, Rotterdam, The Netherlands; 8 Faculty of Medicine, Pontificia Universidad Javeriana, Bogotá, Colombia

**Keywords:** Attitude to health, breast neoplasms, cancer, early detection of cancer, mass screening, oncology, patient acceptance of healthcare, patient participation, systematic review

## Abstract

**Objective:**

To assess the determinants of the participation rate in breast cancer screening programs by conducting a systematic review of reviews.

**Methods:**

We conducted a systematic search in PubMed via Medline, Scopus, Embase, and Cochrane identifying the literature up to April 2019. Out of 2258 revealed unique abstracts, we included 31 reviews, from which 25 were considered as systematic. We applied the Walsh & McPhee Systems Model of Clinical Preventive Care to systematize the determinants of screening participation.

**Results:**

The reviews, mainly in high-income settings, reported a wide range for breast cancer screening participation rate: 16–90%. The determinants of breast cancer screening participation were simple low-cost interventions such as invitation letters, basic information on screening, multiple reminders, fixed appointments, prompts from healthcare professionals, and healthcare organizational factors (e.g. close proximity to screening facility). More complex interventions (such as face-to-face counselling or home visits), mass media or improved access to transport should not be encouraged by policy makers unless other information appears. The repeated participation in mammography screening was consistently high, above 62%. Previous positive experience with screening influenced the repeated participation in screening programs. The reviews were inconsistent in the use of terminology related to breast cancer screening participation, which may have contributed to the heterogeneity in the reported outcomes.

**Conclusions:**

This study shows that consistent findings of systematic reviews bring more certainty into the conclusions on the effects of simple invitation techniques, fixed appointments and prompts, as well as healthcare organizational factors on promoting participation rate in screening mammography.

## Background

A recent meta-analysis of randomized controlled trials showed that an increase in 1% of the participation rate in screening mammography led to a statistically significant 3% reduction in advanced stage and death from breast cancer.^1^ Furthermore, high participation influences program efficiency because the resources invested to launch and maintain a screening program may not be justified if the screening is not acceptable to the target population groups. Thus, participation in breast cancer screening (BCS) has been recently gaining significant importance in the evaluation and implementation of organized screening programs.

Participation in screening programs can be partially explained by behavioural theories targeting to understand and amend human behaviours. Many of these theories limit the scope of promotion strategies to individual cognition, ignoring environmental or economic factors that may influence a person’s intention to participate in screening.^2^ Alternatively, several planning models with a focus on socio-ecological factors and health systems have been applied in promoting BCS. Among them, Walsh and McPhee’s Systems Model of Clinical Preventive Care^3^ was purposely developed to be broad enough to encompass different preventive activities.

Walsh & McPhee’s Systems Model is a comprehensive theoretical framework structuring the determinants of preventive health behavioural changes with a focus on patient–physician interaction. Both the patient and the physician contribute to the development of preventive behaviour, while each entity separately is influenced by a set of predisposing (e.g. sociodemographics, beliefs), enabling (e.g. abilities), and reinforcing (e.g. rewards) factors. In addition, external factors influence preventive behaviours, such as healthcare delivery (i.e. access to care), preventive (i.e. characteristics of the preventive activity itself), and situational (i.e. triggers to health behaviour) factors. Walsh & McPhee’s Systems Model contains components of behavioural, communication, health education, and psychosocial theories and has been applied successfully to explain behaviour in multiple preventive programs including cancer screening.^4–6^

We apply the Walsh & McPhee Systems Model in structuring the overview of the reviews. The research design aims to advise decision-makers on the best evidence they need in the pool of multiple available systematic reviews. While systematic reviews of original evidence are focused on narrow well-defined questions, a review of reviews allows using higher quality evidence, leading to better decision making by critically appraising and combining the results of different secondary analyses. No study, to our knowledge, has ever analysed the results from systematic reviews on participation in BCS programs among the general population. Therefore, the primary purpose of this review is to provide a broad synthesis of contributing factors to participation rates in BCS via mammography under the theoretical framework described. The secondary objective of the review is to evaluate the quality of the systematic reviews reporting participation rates in BCS programs and the degree of consistency in the terminology being used.

## Methods

### Design and search strategy

The design of this study was reported in the published protocol,^7^ registered in PROSPERO (CRD42016050764). We searched systematically the databases PubMed via Medline, Scopus, Embase, and Cochrane, and the grey literature (supplementary Appendix A) with the last update in May 2019. The search line developed for PubMed and adapted for the other databases was modified from the search strategy of Bonfill et al.^8^ (the details are presented in supplementary Appendix A). In our review, we included reviews from commencement until the search date on mammography screening or multiple BCS approaches including mammography screening as one of the target interventions among the general population.

### Search outcomes

Participation rate in BCS, the primary outcome of the study, was defined as the number of women who have a screening test as a proportion of all women who are invited to attend for screening.^9^ This definition was mainly referred to as “participation” or “uptake” in the literature.^9–11^ A secondary outcome that we used was attendance or coverage. Coverage is defined by the European Guidelines for Quality Assurance in Breast Cancer Screening and Diagnosis as the percentage of the target population undergoing screening.^9^ Since coverage represents the availability of screening rather than preferences, we report it as a secondary outcome, aiming to compare the terminologies and definitions applied in the systematic reviews on BCS.

Considering opacity in definitions used in secondary analyses of evidence, we pre-established the following terminology to summarize the reviews’ findings:

- Participation rate – when talking about reviews of studies of any design when it is defined as so, and for the reviews of all randomized controlled trials, controlled trials, and quasi-experimental designs without definition.
- Attendance rate – when talking about reviews of studies of any design when using the definition of coverage or an equivalent, and for all results from mixed or observational studies without definition.

To assess the factors contributing to the participation rate, we applied the Walsh & McPhee Systems Model of Clinical Preventive Care.^3^ Specifically, we grouped the factors identified as predisposing, enabling, reinforcing, organizational, situational, and preventive.

### Data extraction and synthesis

Both abstracts and full texts were screened in duplicate. All the data were extracted using a pre-tested data extraction form with single-entry by the first author (OM) and verified by a second reviewer (NZ, FM, COR, JS or RM). If the participation rate was not reported, we attempted to calculate it from the reviews or summaries of the original data. Considering high heterogeneity in methods and outcomes, we applied structured qualitative synthesis.

### Quality assessment

Two authors assessed independently the quality of the reviews with the Assessing the Methodological Quality of Systematic Reviews (AMSTAR) checklist,^12^ solving any disagreements by consensus. Because discriminating the reviews’ outcomes by quality may lead to biased conclusions, we included all the reviews independently on their quality score. Meanwhile, we excluded from summaries the reviews scored two or less on AMSTAR, considering them as non-systematic.

## Results

**Figure 1.**
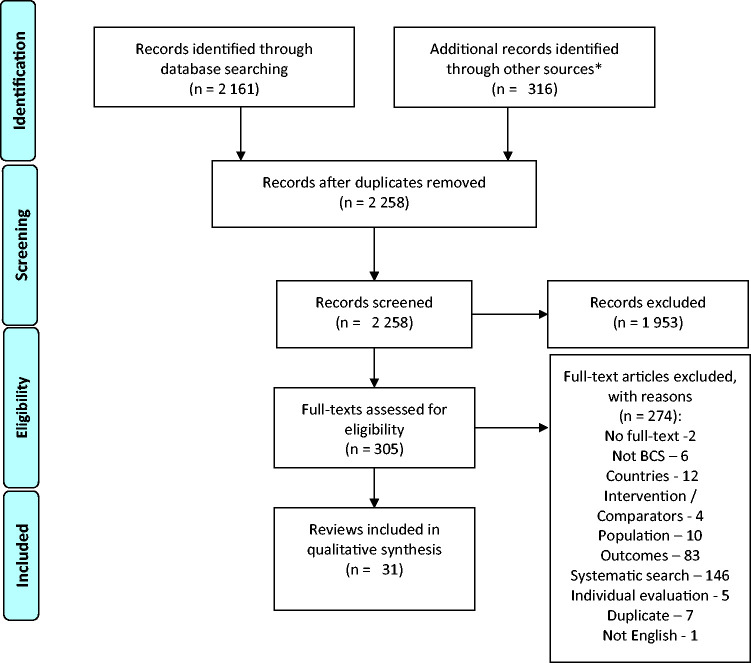
PRISMA 2009 flow diagram used for breast cancer screening review. *Supplementary search. See Moher et al.^30^

We identified 2161 abstracts through systematic and 316 more through non-systematic search, resulting in 31 included reviews (Figure 1), of which 25 were considered systematic. The inter-rater reliability between the two reviewers for the decisions on full-texts inclusion was 85% (Cohen’s kappa = 0.63, substantial agreement). Although the search was not limited to English-language publications, we excluded one of the reviews based on this criterion deviating from the protocol (supplementary Appendix B). The characteristics of the included reviews are presented in supplementary Appendix C.

The systematic reviews had a broad geographic perspective (supplementary Appendix C), with some of them aiming to identify an evidence relevant for either specific countries (USA, the UK, France, Canada, or Japan),^13–20^ geographic regions,^1^ or populations by income or ethnicity.^21–24^ Most of the reviews focused on interventions to improve participation rate (14) or behaviour of screened population and associated factors (12). The majority of reviews were publicly funded and no reviews reported a private source of funding (supplementary Appendix C).

**Figure 2.**
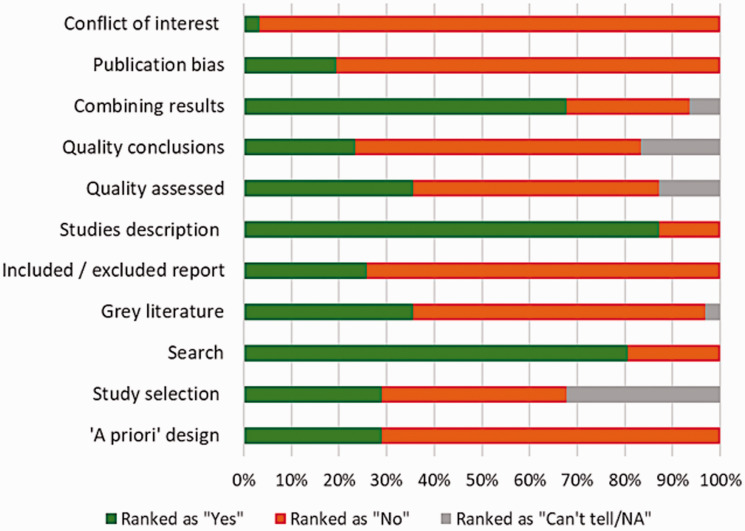
Quality of systematic reviews graded by AMSTAR criteria.

We did not find any difference in the conclusions of the reviews based on their AMSTAR score, date of search or date of publication. The AMSTAR quality criteria that were the least frequently fulfilled (Figure 2) were those related to reporting the excluded studies (eight or 26% of the reviews), formulating conclusions on the basis of the scientific quality of the included studies (seven or 23%), assessing the likelihood of publication bias (six or 19%), and reporting conflict of interest for the included studies (one study). Other limitations of the reviews included a lack of clarity on the first or successive calls for screening uptake, geographical origin of the included original evidence, and weaknesses in methodologic designs of original studies (supplementary Appendix D). Only 22 out of the 31 reviews were at least mentioning the limitations of the original studies. These included lack of theory use,^24^ quality of methodology/design,^1,15–18,20,24–27^ poor reporting of the methods or terminology,^19,20,28^ ethical issues,^18^ and limited generalizability particularly since very few studies were conducted outside of the USA^29^ (supplementary Appendix D).

### Participation in mammography screening

BCS attendance within short timeframe (<5 years) ranged 15–92% in systematic and 1–92% in all the reviews; the participation rate was 6–90% in systematic and narrative reviews and 40–90% among the women invited to screening in the reviews of randomized controlled trials exploring breast cancer mortality decrease with mammography screening (supplementary Appendix E). Only one review based on randomized trials defined the target outcomes as “number of attendees divided by the number of invitees”; this review showed less variable participation range (61–90%).^1^

Secondary attendance to mammography screening (re-attendance) was consistently high, above 62% in four reviews.^15,16,28,31^ Non-attenders of the first visit had a much lower attendance rate after reminders were sent to them than in the cohort of women who responded positively to their first screening invitation (4–42% vs. 46–86%).^13^ The re-attendance was higher with biennial than annual screening in the review by Vernon et al.,^28^ and was lower among those women who previously experienced pain during mammography according to Whelehan et al., though these results were not statistically significant.^31^

Overall, the reviews were inconsistent regarding the terminology or the definitions of the outcomes: some reviews did not define the outcomes used, whereas others combined in one synthesis “coverage” and “participation rates” (supplementary Appendix E).

### Participation in other BCS programs

Only a few reviews reported population preferences for other BCS approaches. A narrative review reported a participation rate in clinical breast examination ranging between 13 and 70% among women in Arabic countries,^32^ while another one reported 31 to 72%.^27^ However, the outcomes in both reviews were not clearly defined. No reviews reported participation rates when using BCS ultrasonography.

### Regional variabilities in BCS participation and attendance

The reviews included studies mainly from high-income Western or North American countries. Most of the evidence was coming from the USA (supplementary Appendix C), with three reviews being able to identify studies only from this country.^22,25,26^ A few reviews targeting Asian populations^1,17,24^ included European and North American trials and therefore did not detect regional variabilities. While there are no sufficient data describing regional differences, Brewer et al.^16^ and Bhargava et al.^23^ reported variability in geographical regions (supplementary Appendix E);^16,23^ for instance, re-attendance rate was higher in Western Europe: 71–94% vs. 57–81% in the USA or 49–74% in Canada.^16^ Few reviews included information on low- and middle-income countries (Chile, South Africa, Brazil, Thailand, and Mexico)^21,33^ and reported only generalized data. Therefore, it is difficult to make any definitive conclusions regarding the outcomes in such settings.

### Factors contributing to variability in BCS participation and attendance

**Figure 3.**
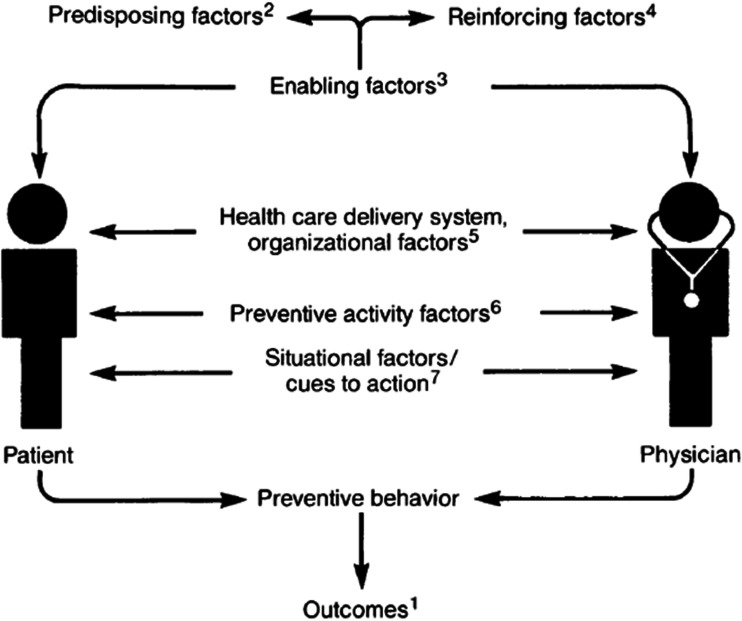
The systems model of clinical preventive care by Walsh and McPhee. (1) Outcomes are defined as decreased disease incidence, decreased morbidity, and decreased mortality. (2) Predisposing factors relate to the motivation to perform a particular health behaviour. (3) Enabling factors include the skills and resources necessary to perform the behaviour. (4) Reinforcing factors are those that support or reward the behaviour. (5) Healthcare delivery system/organizational factors include access to care; availability of technology and personnel; organizational priorities; structure of office practice; reimbursement; and coordination with community resources. (6) Preventive activity factors are features of the preventive activity itself and include costs; risks; efficacy; and effectiveness. (7) Situational factors/cues to action are triggers to health behaviour and include internal cues, such as symptoms and external cues such as physician reminders (Reproduced with permission from Walsh and McPhee^3^).

We examined the impact of multiple factors on mammography screening participation, attendance, and re-attendance, using the Walsh & McPhee Systems Model (Figure 3).^3^ In general, the reviews based on randomized trials are more oriented to factors related to program organization and the process of invitation, while reviews based on observational studies analyse more contextual factors such as demographic and cultural barriers, corresponding to those including countries from regions other than North America and Europe (supplementary Appendices C, E).

We summarize factors defining participation and attendance rates where the original and secondary evidence were consistent in Table 1, and those with inconsistent effect in Table 2. Overall, the reviews were inconsistent regarding an impact of patient’s predisposing factors on getting a future screening mammogram, in particular whether other preventive behaviours, medical history, demographic description, or self-beliefs and worries could affect women’s decision to attend BCS (Table 2). For instance, the reviews were inconsistent as to whether higher uptake rate was associated with young age,^14,15,24^ ethnicity and culture,^14,23,34^ or socio-economic factors, defined by deprivation area, levels of education, and income.^14,15,21^ Jepson et al. found that high level of education was significant only in 17% of studies and population ethnicity in 33% of the studies, with income, religion, and language spoken also defined as insignificant variables.^14^ Soler-Michel et al. discussed whether socio-economic characteristics were not important because of heterogeneity in data depending on jurisdictions included (e.g. no impact of socio-economic characteristics in Finland, Italy or France).^15^ However, the meta-analysis of Damiani et al.^21^ found that women with the highest level of education were more likely to be screened, even though not every study included reached significance on this indicator. In general, the reviews that concluded on questionable impact of socio-economic determinants were older (searched before 2003) and relied on both cross-sectional and prospective studies, whereas the positively framed reviews were based on more recent cross-sectional evidence.^14,15,23,24^

**Table 1.** Factors with a consistent direction of effect on participation rate in BCS.

Factors	Direction of effect	Frequency or reporting/source
Patient predisposing factors		
Intentions to screening	Positive	Two reviews^14,15^
Patient enabling factors		
Removal of financial barriers	Positive	Four reviews^14,15,22,29^ vs. 1 unclear effect^20^
Simple letters or calls	Positive	Six reviews^8,14,19,22,29,34^
Tailoring by ethnicity	No or limited effect	One review^35^
“Word of mouth”^a^	Positive	One review^32^
Recommendation/support by a healthcare provider	Positive	Three reviews^14,19,35^
Small media	Positive	One review^20^
Big media/social networks	No or limited effect	Three reviews^13,20,34^
Extensive written or verbal health education vs. brief recommendations	No or limited effect	Three reviews^13,18,22^
Telephone counselling	Positive	Four reviews^8,14,28,29^
Home visits vs. no visits	Positive	Two reviews^14,29^
Home visits vs. simple invitation	No or limited effect	Two reviews^8,13^
Individual education	Positive	Four reviews^18,20,22,28^
Healthcare delivery system/organizational factors		
Organizational features of a healthcare system	Positive	One review^29^
Opportunistic screening or low coverage rate^b^	Positive	Two reviews^14,29^
Health insurance	Positive	Two reviews^14,24^
Structural reorganizations	Positive	Three reviews^14,20,22^
Management systems	Positive	Three reviews^14,20,22^
Mobile mammography or community screening	Positive	Three reviews^14,25,33^
Test/preventive activity factors		
Previous mammography	Positive or negative	Four reviews^14–16,36^
Situational factors/cues to action		
Risk-factor questionnaires	No or limited effect	Two reviews^14,26^
Fixed appointment with any invitation approach	Positive	Four reviews^13,14,18,22^
Reminder letters or invitation follow-ups	Positive	Eight reviews^8,13,14,18,19,22,28^ vs. one unclear effect^20^
Phone reminders	No or limited effect	One review^13^
Endorsement by general practitioner	No or limited effect	One review^13^
Second mailed	Positive	One review^34^
Provider’s prompts	Positive	Four reviews^14,18,19,28^

^a^Information received from family or friends.

^b^Before intervention.

**Table 2.** Factors with unclear or undefined effect on participation rate in BCS.

Factors	Level of disagreement	Source
Patient predisposing factors		
Medical history	Inconsistent in reviews	^14,15,36^
Other preventive behaviour		^14,15^
Self-beliefs and worries		^14,15,24,29^
Demographic factors		^14,15,21,23,24,28^
Patient enabling factors		
Knowledge of breast cancer and/or BCS	Inconsistent in reviews	^14,15,33^
Tailoring vs. personalized invitation	Unclear original evidence	^18,22^
Public information campaigns	Unclear original evidence	^18,20^
Face-to-face counselling	Inconsistent in reviews	^14,18,20,22,37^
Printed information materials in addition to standard invitation	Unclear original evidence^14,18,20,35^ and inconsistent in reviews	^8,14,18,20,34^
Academic detailing or use of theory	Unclear original evidence	^22,28^
Group education	Unclear original evidence	^20^
Patient reinforcing factors		
Rewards or incentives to patients	Unclear original evidence^18,20,34^ and inconsistent in reviews	^14,18,20,34^
Provider reinforcing factors		
Rewards or incentives to providers	Unclear original evidence	^20^
Physician enabling factors		
Physician education	Inconsistent in reviews	^14,22^
Healthcare delivery system/organizational factors		
Settings and screening approaches (e.g. intervals)	Unclear original evidence	^28^
Multistrategy examinations	Inconsistent in reviews	^8,13,14,28^
Reduction of logistical barriers/transport access	Unclear original evidence^13^ and inconsistent in reviews	^13,15^
Facilitated appointment schedule	Inconsistent in reviews	^15,22^
Situational factors/cues to action		
Face-to-face reminder	Unclear original evidence	^18,20^

BCS: breast cancer screening program.

Intentions to screening was the only consistent patient’s predisposing factor associated with BCS attendance (Table 1).^14,15^ Physician predisposing factors (demographics, gender, ethnicity, language, beliefs, attitudes, prior clinical experience, and personal health practices) were not reported in the summarized literature.

The enabling factors of Walsh & McPhee’s Systems Model^3^ are related to skills and resources necessary to perform the behaviour. In this group, we included the elimination of financial barriers, general knowledge of breast cancer risk and BCS, approaches to information and invitation delivery, access to screening, and approaches towards education about BCS.

The reviews agreed on the importance of financial barriers in patients’ screening decisions,^14,15,22,33^ while Brouwers et al.^20^ concluded on insufficient evidence to recommend for or against reducing out-of-pocket costs related to mammography screening.^20^ The reviews were consistent that simple active recruitment strategies, such as letters of invitation or phone calls, improve participation (Table 1).^8,14,19,22,29,35^ While interventions promoted higher participation among ethnic minorities including the use of bilingual instructions, and patients’ handouts or forms,^22^ tailoring invitations to ethnicity had a negative impact on screening participation.^35^ Interestingly enough, there was no consistency in the reviews on importance of screening knowledge;^14,15,24^ the evidence was unclear whether tailored invitation interventions in general are more effective than standard ones.^18,22^ Nevertheless, the reviews agreed that untargeted mass invitations (including social networks and mass media) had no effect on screening participation,^13,20,34^ while recommendations from healthcare providers were important.^14,19,35^

Regarding education-related interventions, the reviews based on both trials and observational evidence showed a positive impact of telephone counselling,^8,14,28,29^ face-to-face counselling,^18,20,22,28^ and educational home visits^14,29^ on uptake of screening mammography. However, the last two types of intervention were non-superior to simple calls or letters.^8,13,14,35^ In addition, three reviews found no evidence of a positive effect, and even a negative impact, of very extensive health information on screening participation directed toward women of low educational background.^13,18,22^

Physician enabling factors include training, technical expertise, knowledge, and resources.^3^ Among these factors, only physician education was assessed in two reviews, concluding on no or unclear effect on BCS participation/attendance.^14,22^

Reinforcing factors of the Walsh & McPhee Systems Model,^3^ such as rewards or incentives, were limitedly reported in the included literature. Both patient^14,18,20,34^ and provider^20^ reinforcing factors were not determined as evidently consistent in the reviews.

The most frequently reported healthcare delivery system organizational factors were logistic, structure, and management related. A short distance to facilities (for instance, mobile mammography units or community hospitals) could increase access to BCS and ultimately increase participation rates.^14,25,33^ Meanwhile, improved logistics, such as access to bus transportation, did not have the same strengths of evidence.^13,15^

In terms of preventive activity factors, the reviews showed that they were related to previous positive or negative experiences of mammography.^14–16,31^ Women who had a positive experience indicated an improved re-attendance,^15^ while those who experienced pain during the past mammography and a related anxiety had a lower attendance rate.^15,31^ The direction of effect of previous false-positive results of mammographic screening on attendance rate in subsequent screenings is not straightforward and consistent between different geographic regions as shown in the review by Brewer et al.^16^

Finally, in regard to the situational factors and cues to action, fixed appointment with any invitation approach^13,14,18,22^ as well as mailed patient reminders^8,13,14,18,19,22,28^ were reported as highly effective to increase participation. Curbow et al.^34^ also commented on the potential effectiveness of second mailed reminders. Similarly, providers’ prompts or reminders were considered to be effective.^14,18,19,28^

On the other hand, insufficient evidence was reported on clear differences among the different reminder strategies (for example, between a face-to-face reminder and a telephone call),^18,20^ and there were conflicting conclusions in proposing convenient appointment scheduling for women invited to screening.^15,22^ In addition, reviews showed that risk-factor questionnaires had no effect on BCS.^14,26^

## Discussion

Our review of reviews highlighted several important issues. Firstly, it identified factors with a consistent impact on BCS participation rate, allowing policy makers to focus on interventions with little or no uncertainty regarding a positive impact. For women invited to screening for the first time, such interventions with a positive effect include:

- Removal of financial barriers
- Simple invitation approaches (e.g. invitation letter)
- Healthcare providers’ recommendations
- Organizational factors related to healthcare system delivery
- Fixed appointments
- Reminders and providers’ prompts.

Since re-attendance is affected by women’s previous screening experience, screening providers may use the observed relief effect (more positive perception of screening shortly after the procedure)^37^ to address any distress related to mammography immediately after the procedure in order to improve long-term memories of BCS.

Secondly, our review demonstrated a possible inconsistency in the reviews’ conclusions, putting into question the a priori perception of the reviews as a “lens of evidence” in the hierarchy of the pyramid of evidence. The findings of the different reviews vary depending on the study type (e.g. intervention vs. observational), the country where the evidence was obtained, type of data synthesis approach (qualitative or quantitative), and the year limits in the search strategy. Our review found inconsistent results regarding socioeconomic conditions as a determinant of participation in BCS, possibly due to the types of studies included in the reviews, years of search, and data analysis applied. Indeed, some population-based research demonstrate a link between socio-economic characteristics and cancer disparities,^38–41^ as well as screening participation and deprivation.^42^ However, a recent pooled cross-sectional time series analysis of 17 European countries with established organized BCS programs did not find an association between participation rate in BCS and socio-economic characteristics.^43^ This study supports our observations on geographic differences in screening outcomes. Moreover, it emphasizes that certain questions specific to the context of geographical jurisdictions (e.g. relationships between socio-economic variables and participation rate) should be informed not only by the pooled summaries but also by the national statistical data. Intervention studies show that removal of health system and financial barriers is positively associated with participation, and cancer disparities being reduced through organized screening,^43–46^ although these efforts are not equally effective in all the jurisdictions.

Thirdly, by looking at disagreements between the reviews, our study identified the areas with possibly a high value of information. Ambiguity exists in relationships between the BCS participation rate and demographic characteristics of the population, knowledge of breast cancer and screening, face-to-face counselling, physicians’ education, multi-strategy examinations, and facilitated appointments. Taking into account the limitations of the included secondary analyses, a high-quality comprehensive review of relationships between indicators of socio-economic determinants and screening uptake, comparing the possible differences among jurisdictions, will be an asset to conclude on the raised contradictions.

Fourthly, our study revealed differences between reviews related to definitions used which could lead to possible confusion in terminology, for example between participation rate and attendance.^19,22,27,33^ Such inconsistencies in measures and definitions of screening participation are common in reviews of other cancer screening programs.^10^ The definitions of the European guidelines for quality assurance in BCS and diagnosis for “participation rate,” “coverage by invitation,” and “coverage by screening”^15^ should be reinforced to follow globally, to avoid confusion in the assessment, reporting, and interpretation of BCS participation.

### Study limitations

Given the large scope of this systematic review of reviews, we may have missed some of the literature related to the topic, despite the attempt to identify all relevant data. We also note the limitations of using AMSTAR for judging the quality of the reviews on screening participation rate; for example, the questions on data synthesis used in AMSTAR may not be directly relevant for reviews of non-clinical outcomes. Furthermore, the reviews could have other limitations not highlighted by AMSTAR, such as incorrect or unclear reporting of the target outcome. As such, a high quality score on AMSTAR may not mean an absence of bias in the systematic reviews that were evaluated.

### Clinical policy implications

Since implementation of BCS programs has already stimulated multiple discussions about their benefit/harm ratio and cost-effectiveness,^47,48^ understanding how much support these programs gain from various groups, such as the medical community and the women themselves, can be an important parameter defining the programs’ priority on the political agenda. This is especially crucial for countries considering implementation of a population-wide screening program, re-assessing continuation of the existing program, or evaluating screening extension to other age groups. Moreover, knowledge of the determinants of BCS participation would help to design programs receiving higher acceptance from the affected groups and could improve implementation outcomes. As suggested by this study, developing policies such as scheduling fixed appointments while inviting the women to screening, or requesting that medical providers prompt women regarding screening mammograms, can boost participation rates. Besides, simple population-oriented strategies, and system and structural interventions are effective to increase participation in mammography screening and should be considered by program commissioners.

## Conclusions

While systematic reviews are perceived as a “lens of evidence,” their results are not always consistent. Policy makers should prioritize the interventions consistently reported as effective in secondary research, and critically assess the applicability of the review findings to the local context.

## Supplemental Material

sj-pdf-1-msc-10.1177_0969141320930743 - Supplemental material for Systematic reviews as a “lens of evidence”: Determinants of participation in breast cancer screening Click here for additional data file. Supplemental material, sj-pdf-1-msc-10.1177_0969141320930743 for Systematic reviews as a “lens of evidence”: Determinants of participation in breast cancer screening by O Mandrik, E Tolma, N Zielonke, F Meheus, C Ordóñez-Reyes, JL Severens and R Murillo in Journal of Medical Screening
