# Supplementary material for: Systematic reviews as a “lens of evidence”: Determinants of participation in breast cancer screening
Source: J Med Screen. 2020 Jun 9;28(2):70–9. doi: 10.1177/0969141320930743 (PMC8167916; doi:10.1177/0969141320930743)
Supplement: sj-pdf-1-msc-10.1177_0969141320930743 - Supplemental material for Systematic reviews as a “lens of evidence”: Determinants of participation in breast cancer screening [file sj-pdf-1-msc-10.1177_0969141320930743.pdf]

Appendices

Appendix A. Search strategy

Pubmed via Medline (adapted for Scopus and Embase accordingly):

(((PATIENT-ACCEPTANCE-OF-HEALTH-CARE*:ME) OR REMINDER-SYSTEMS*:ME) OR APPOINTMENTS-AND-SCHEDULES*:ME) OR CORRESPONDENCE*:ME) OR MOBILE-HEALTH-UNITS*:ME) OR PREVENTIVE-HEALTH-SERVICES*:ME) OR (HEALTH-PROMOTION*:ME)) OR (letter*[Title/Abstract] OR mail*[Title/Abstract] OR phone*[Title/Abstract] OR telephone*[Title/Abstract] OR invit*[Title/Abstract] OR send*[Title/Abstract] OR sent*[Title/Abstract] OR attendan*[Title/Abstract] OR appointmen*[Title/Abstract] OR recruitm*[Title/Abstract] OR coverag*[Title/Abstract] OR usag*[Title/Abstract] OR participat*[Title/Abstract])) OR adherence OR preferences OR compliance OR uptake OR attendance) AND ((breast[Title/Abstract] AND cancer[Title/Abstract]) AND (("screening"[All Fields] OR "mass screening"[MeSH Terms] OR ("mass"[All Fields] AND "screening"[All Fields]) OR "mass screening"[All Fields] OR "screening"[All Fields] OR "early detection of cancer"[MeSH Terms] OR ("early"[All Fields] AND "detection"[All Fields] AND "cancer"[All Fields]) OR "early detection of cancer"[All Fields]) OR ("mammography"[MeSH Terms] OR "mammography"[All Fields]) OR "ultrasonography"[All Fields] OR "ultrasonography"[MeSH Terms]) OR (Clinical[All Fields] AND ("breast"[MeSH Terms] OR "breast"[All Fields]) AND ("physical examination"[MeSH Terms] OR ("physical"[All Fields] AND "examination"[All Fields]) OR "physical examination"[All Fields] OR "examination"[All Fields])) OR ("breast self-examination"[MeSH Terms] OR ("breast"[All Fields] AND "self-examination"[All Fields]) OR "breast self-examination"[All Fields] OR ("self"[All Fields] AND "breast"[All Fields] AND "examination"[All Fields]) OR "self breast examination"[All Fields])) AND (Review[ptyp] AND "humans"[MeSH Terms])

Cochrane Database of Systematic Reviews (first 300 hits): Breast cancer AND screening

Additional search including the grey literature:

Journals: Plos One, Cancer, Cancer Epidemiology, Breast, Breast Cancer, Journal of Surgical Oncology, Lancet Oncol., JAMA

Data bases: Google Scholar (“breast cancer screening effect review”)

Reports and other grey literature: Blue Cross and Blue Shield Association; The National Institute for Health and Care Excellence (NICE), The Canadian Agency for Drugs and Technologies in Health (CADTH, Canada), Institute for Quality and Efficiency in Health Care (IQWIG, Germany), NHS Quality Improvement Scotland; Canadian Coordinating Office for Health Technology Assessment (CCOHTA); Comite d’Evaluation et de Diffusion des Innovations Technologiques (CEDIT); Agence Nationale d'Accréditation et d'Evaluation en Santé (ANAES); the American Society of Clinical Oncology (ASCO), the American Cancer Society, the International Agency for Research on Cancer, and the European Society for Medical Oncology.

Appendix B. Excluded reviews grouped by the primary reason for the exclusion

1. Full text not available

1. Katzen J, Dodelzon K. A review of computer aided detection in mammography. Clinical Imaging. 2018;52:305-9.
2. Yabroff, K.R., et al., *Inreach and outreach interventions to improve mammography use.* J Am Med Womens Assoc (1972), 2001. **56**(4): p. 166-73, 188.

2. Not focused on breast cancer screening

1. Ganschow, P.S., et al., *Update in women's health.* J Gen Intern Med, 2009. **24**(6): p. 765-70.
2. Hashemi SM, Balouchi A, Al-Mawali A, Rafiemanesh H, Rezaie-Keikhaie K, Bouya S, et al. Health-related quality of life of breast cancer patients in the Eastern Mediterranean region: a systematic review and meta-analysis. Breast Cancer Research and Treatment. 2019;174(3):585-96. PubMed PMID: 625903157.
3. Leinweber KA, Columbo JA, Kang R, Trooboff SW, Goodney PP. A Review of Decision Aids for Patients Considering More Than One Type of Invasive Treatment. Journal of Surgical Research. 2019;235:350-66. PubMed PMID: 2001277555.
4. Nelson, H.D., et al., *Risk factors for breast cancer for women aged 40 to 49 years: a systematic review and meta-analysis.* Ann Intern Med, 2012. **156**(9): p. 635-48.
5. Snell, J.L. and E.L. Buck, *Increasing cancer screening: a meta-analysis.* Prev Med, 1996. **25**(6): p. 702-7.
6. Quante AS, Strahwald B, Fischer C, Kiechle M. Individualized risk of breast cancer-How should it be calculated, evaluated and discussed?. [German]. Gynakologe. 2018;51(5):397-402. PubMed PMID: 621917435.

3. Conducts search only for one country

1. Bobdey, S., et al., *Cancer screening: Should cancer screening be essential component of primary health care in developing countries?* International Journal of Preventive Medicine, 2015. **2015**(July).
2. Bond, M., et al., Systematic review of the psychological consequences of false-positive screening mammograms. Health Technol Assess, 2013. 17(13): p. 1-170, v-vi.
3. Chan, D.N. and W.K. So, *A systematic review of randomised controlled trials examining the effectiveness of breast and cervical cancer screening interventions for ethnic minority women.* Eur J Oncol Nurs, 2015. **19**(5): p. 536-53.
4. Clark, M.A., W. Rakowski, and L.B. Bonacore, *Repeat mammography: prevalence estimates and considerations for assessment.* Ann Behav Med, 2003. **26**(3): p. 201-11.
5. Escoffery, C., et al. A systematic review of special events to promote breast, cervical and colorectal cancer screening in the United States (Provisional abstract). BMC Public Health, 2014. 14, 274.Larsson, L.G., *Controversies in screening with mammography.* Acta Oncologica, 1997. **36**(7): p. 675-679.

6. Khan-Gates, J.A., et al., Geographic Access to Mammography and Its Relationship to Breast Cancer Screening and Stage at Diagnosis: A Systematic Review. Womens Health Issues, 2015. 25(5): p. 482-93.

7. Mandelblatt, J.S. and K.R. Yabroff, *Effectiveness of interventions designed to increase mammography use: a meta-analysis of provider-targeted strategies.* Cancer Epidemiol Biomarkers Prev, 1999. **8**(9): p. 759-67.

8. Majidi, A., et al., Cancer screening awareness and practice in a middle income country; A systematic review from Iran. Asian Pacific Journal of Cancer Prevention, 2017. 18(12): p. 3187-3194.

9. Meissner, H.I., et al., *Breast and cervical cancer screening interventions: an assessment of the literature.* Cancer Epidemiol Biomarkers Prev, 1998. **7**(10): p. 951-61.

10. Otto, S.J., et al., *Initiation of population-based mammography screening in Dutch municipalities and effect on breast-cancer mortality: a systematic review.* Lancet, 2003. **361**(9367): p. 1411-7.

11. Scheel, J.R., et al., *Screening ultrasound as an adjunct to mammography in women with mammographically dense breasts.* Am J Obstet Gynecol, 2015. **212**(1): p. 9-17.

12. Secginli, S., et al., Interventions Promoting Breast Cancer Screening Among Turkish Women With Global Implications: A Systematic Review. Worldviews on evidence-based nursing, 2017. 14(4): p. 316-323.

4. Other interventions/comparators

1. Abadir, A.M., et al., *Influence of qualitative research on women's health screening guidelines.* Am J Obstet Gynecol, 2014. **210**(1): p. 44.e1-6.

2. Akbari Sari, A., M. Mobinizadeh, and M. Azadbakht, *A systematic review of the effects of diffuse optical imaging in breast diseases.* Iran J Cancer Prev, 2013. **6**(1): p. 44-51.

3. Cummings, S.R., et al., *Prevention of breast cancer in postmenopausal women: approaches to estimating and reducing risk.* J Natl Cancer Inst, 2009. **101**(6): p. 384-98.

4. Noble, M., et al., *Computer-aided detection mammography for breast cancer screening: systematic review and meta-analysis.* Arch Gynecol Obstet, 2009. **279**(6): p. 881-90.

5. Other population

1. *ACR practice guideline for the performance of screening and diagnostic mammography.* 2013, American College of Radiology.

2. Andresen, E.M., et al., *Pap, mammography, and clinical breast examination screening among women with disabilities: a systematic review.* Womens Health Issues, 2013. **23**(4): p. e205-14.

3. Arasu VA, Kannan N, Krishnarao PM, Kuehner G, Kuan MC, Kim JC, et al. Imaging the Breast in Pregnant or Lactating Women. Current Radiology Reports. 2018;6(2). PubMed PMID: 620499133.

4. Houssami N, Cho N. Screening women with a personal history of breast cancer: Overview of the evidence on breast imaging surveillance. Ultrasonography. 2018;37(4):277-87. PubMed PMID: 624498486.

5. Kuhl CK. Abbreviated breast MRI for screening women with dense breast: The EA1141 trial. British Journal of Radiology. 2018;91(1090). PubMed PMID: 624320747.

6. Lamontagne-Godwin F, Burgess C, Clement S, Gasston-Hales M, Greene C, Manyande A, et al. Interventions to increase access to or uptake of physical health screening in people with severe mental illness: A realist review. BMJ Open. 2018;8(2). PubMed PMID: 623793851.

7. Nothacker, M., et al., *Early detection of breast cancer: benefits and risks of supplemental breast ultrasound in asymptomatic women with mammographically dense breast tissue. A systematic review.* BMC Cancer, 2009. **9**: p. 335.

8. Schouten B, Avau B, Bekkering GE, Vankrunkelsven P, Mebis J, Hellings J, et al. Systematic screening and assessment of psychosocial wellbeing and care needs of people with cancer. Cochrane Database of Systematic Reviews. 2019;2019(3). PubMed PMID: 626892156.

9. Smetana GW, Elmore JG, Lee CI, Burns RB. Should this woman with dense breasts receive supplemental breast cancer screening?: Grand rounds discussion from beth Israel deaconess medical center. Annals of Internal Medicine. 2018;169(7):474-84. PubMed PMID: 624155557.

10. Warner E. Screening BRCA1 and BRCA2 mutation carriers for breast cancer. Cancers. 2018;10(12). PubMed PMID: 625405204.

6. Other outcomes

1. Andersen, S.B., et al., A simple way to measure the burden of interval cancers in breast cancer screening. BMC Cancer, 2014. 14: p. 782.

2. Anderson, B.O. and R. Jakesz, *Breast cancer issues in developing countries: an overview of the Breast Health Global Initiative.* World J Surg, 2008. **32**(12): p. 2578-85.

3. Anderson, B.O., et al., *Breast cancer in limited-resource countries: an overview of the Breast Health Global Initiative 2005 guidelines.* Breast J, 2006. **12 Suppl 1**: p. S3-15.

4. Armstrong, K., et al., Screening mammography in women 40 to 49 years of age: a systematic review for the American College of Physicians. Ann Intern Med, 2007. 146(7): p. 516-26.

5. Bahadursingh, S., et al., *Mammographic screening: Is it relevant to developing countries?* Current Medicine Research and Practice, 2014. **4**(4): p. 168-170.

6. Baker, S., M. Wall, and A. Bloomfield, *Breast cancer screening for women aged 40 to 49 years--what does the evidence mean for New Zealand?* N Z Med J, 2005. **118**(1221): p. U1628.

7. Berry, D.A., et al., *Effect of screening and adjuvant therapy on mortality from breast cancer.* N Engl J Med, 2005. **353**(17): p. 1784-92.

8. Barratt, A.L., et al., *Benefits, harms and costs of screening mammography in women 70 years and over: a systematic review.* Med J Aust, 2002. **176**(6): p. 266-71.

9. Baxter, N., Preventive health care, 2001 update: should women be routinely taught breast self-examination to screen for breast cancer? Cmaj, 2001. 164(13): p. 1837-46.

10. Bevers, T.B., et al., *NCCN clinical practice guidelines in oncology: breast cancer screening and diagnosis.* J Natl Compr Canc Netw, 2009. **7**(10): p. 1060-96.

11. Biesheuvel, C., et al., Effects of study methods and biases on estimates of invasive breast cancer overdetection with mammography screening: a systematic review. Lancet Oncol, 2007. 8(12): p. 1129-38.

12. Biesecker, B.B., M.D. Schwartz, and T.M. Marteau, *Enhancing informed choice to undergo health screening: a systematic review.* Am J Health Behav, 2013. **37**(3): p. 351-9.

13. Bowie, J.V., et al., *A review of breast, cervical, and colorectal cancer screening interventions in older women.* Cancer Control, 2005. **12 Suppl 2**: p. 58-69.
14. Breast Cancer Screening. 2011, Canadian Task Force on Preventive Health Care. p. 138.
15. Brett, J., et al., The psychological impact of mammographic screening. A systematic review. Psychooncology, 2005. 14(11): p. 917-38.
16. Brewer, N.T., T. Salz, and S.E. Lillie, Systematic review: the long-term effects of false-positive mammograms. Ann Intern Med, 2007. 146(7): p. 502-10.
17. Broeders, M., et al., The impact of mammographic screening on breast cancer mortality in Europe: a review of observational studies. J Med Screen, 2012. 19 Suppl 1: p. 14-25.
18. Brown, D.W., et al., *Economic evaluation of breast cancer screening: A review.* Cancer Practice, 1999. **7**(1): p. 28-33.
19. Brown, M.L. and L. Fintor, *Cost-effectiveness of breast cancer screening: preliminary results of a systematic review of the literature.* Breast Cancer Res Treat, 1993. **25**(2): p. 113-8.
20. Burda, B.U., et al., *Quality varies across clinical practice guidelines for mammography screening in women aged 40-49 years as assessed by AGREE and AMSTAR instruments.* J Clin Epidemiol, 2011. **64**(9): p. 968-76.
21. Carter, J.L., R.J. Coletti, and R.P. Harris, Quantifying and monitoring overdiagnosis in cancer screening: a systematic review of methods. Bmj, 2015. 350: p. g7773.
22. Dinnes, J., et al., Effectiveness and cost-effectiveness of double reading of mammograms in breast cancer screening: Findings of a systematic review. Breast, 2001. **10**(6): p. 455-463.
23. Elmore, J.G., et al., Screening for breast cancer. Journal of the American Medical Association, 2005. 293(10): p. 1245-1256.
24. Erpeldinger, S., et al., Is there excess mortality in women screened with mammography: a meta-analysis of non-breast cancer mortality. Trials, 2013. 14: p. 368.
25. Galit, W., M.S. Green, and K.B. Lital, Routine screening mammography in women older than 74 years: a review of the available data. Maturitas, 2007. 57(2): p. 109-19.
26. Gartlehner, G., et al., Adjunct ultrasonography for breast cancer screening in women at average risk: a systematic review. Int J Evid Based Healthc, 2013. 11(2): p. 87-93.
27. Gotzsche, P.C. and O. Olsen, Is screening for breast cancer with mammography justifiable? Lancet, 2000. 355(9198): p. 129-34.
28. Gotzsche, P.C. and M. Nielsen, Screening for breast cancer with mammography. Cochrane Database Syst Rev, 2006(4): p. Cd001877.
29. Gotzsche, P.C. and M. Nielsen, Screening for breast cancer with mammography. Cochrane Database Syst Rev, 2009(4): p. Cd001877.
30. Gøtzsche, P.C., Relation between breast cancer mortality and screening effectiveness: Systematic review of the mammography trial. Danish Medical Bulletin, 2011. 58(3).
31. Gøtzsche Peter, C. and J. Jørgensen Karsten Screening for breast cancer with mammography. Cochrane Database of Systematic Reviews, 2013. DOI: 10.1002/14651858.CD001877.pub5.
32. Green, B.B. and S.H. Taplin, Breast cancer screening controversies. J Am Board Fam Pract, 2003. 16(3): p. 233-41.
33. Harris, R., J. Yeatts, and L. Kinsinger, *Breast cancer screening for women ages 50 to 69 years a systematic review of observational evidence.* Prev Med, 2011. **53**(3): p. 108-14.

34. Henriksen EL, Carlsen JF, Vejborg IMM, Nielsen MB, Lauridsen CA. The efficacy of using computer-aided detection (CAD) for detection of breast cancer in mammography screening: a systematic review. Acta Radiologica. 2019;60(1):13-8. PubMed PMID: 621795911.

35. Ho C, H.D., Warburton R, MacGregor J, Pisano E, Joyce J. , Digital mammography versus film-screen mammography: technical, clinical and economic assessments. Technology report no 30. 2002, Canadian Coordinating Office for Health Technology Assessment: Ottawa.

36. Hofvind, S., et al., False-positive results in mammographic screening for breast cancer in Europe: a literature review and survey of service screening programmes. J Med Screen, 2012. 19 Suppl 1: p. 57-66.

37. Hollingsworth AB. Redefining the sensitivity of screening mammography: A review. American Journal of Surgery. 2019. PubMed PMID: 2001544943.

38. Houssami N. Evidence on Synthesized Two-dimensional Mammography Versus Digital Mammography When Using Tomosynthesis (Three-dimensional Mammography) for Population Breast Cancer Screening. Clinical Breast Cancer. 2018;18(4):255-60.e1. PubMed PMID: 618845181.

39. Howard, M., G. Agarwal, and A. Lytwyn, *Accuracy of self-reports of Pap and mammography screening compared to medical record: a meta-analysis.* Cancer Causes Control, 2009. **20**(1): p. 1-13.

40. Huang, Y., et al., *[Evaluation on the accuracy of high-frequency ultrasound being used in the breast cancer screening program in women from Asian countries: a systematic review].* Zhonghua Liu Xing Bing Xue Za Zhi, 2010. **31**(11): p. 1296-9.

41. Humphrey, L., et al., U.S. Preventive Services Task Force Evidence Syntheses, formerly Systematic Evidence Reviews, in Screening for Breast Cancer. 2002, Agency for Healthcare Research and Quality (US): Rockville (MD).

42. Iared, W., et al., Comparative evaluation of digital mammography and film mammography: systematic review and meta-analysis. Sao Paulo Med J, 2011. 129(4): p. 250-60.

43. Irwig, L., N. Houssami, and C. van Vliet, New technologies in screening for breast cancer: a systematic review of their accuracy. Br J Cancer, 2004. 90(11): p. 2118-22.

44. Irvin, V.L. and R.M. Kaplan Screening mammography & breast cancer mortality: meta-analysis of quasi-experimental studies (Provisional abstract). Database of Abstracts of Reviews of Effects, 2014. e98105.

45. Jørgensen, K.J. and P.C. Gøtzsche, Overdiagnosis in publicly organised mammography screening programmes: systematic review of incidence trends. BMJ (Clinical research ed.), 2009. 339: p. b2587.

46. Kerlikowske, K., et al., *Efficacy of screening mammography: A meta-analysis.* Journal of the American Medical Association, 1995. **273**(2): p. 149-154.

47. Kien, C., et al., *[Comparative effectiveness and safety of screening and counselling interventions conducted by non-physicians and physicians: a systematic review].* Z Evid Fortbild Qual Gesundhwes, 2015. **109**(1): p. 18-27.

48. Koleva-Kolarova, R.G., et al., Simulation models in population breast cancer screening: A systematic review. Breast, 2015. 24(4): p. 354-63.

49. Lee, E.H., et al., The Korean guideline for breast cancer screening. Journal of the Korean Medical Association, 2015. 58(5): p. 408-419.

50. Legler, J., et al., *The effectiveness of interventions to promote mammography among women with historically lower rates of screening.* Cancer Epidemiol Biomarkers Prev, 2002. **11**(1): p. 59-71.

51. Lu, M., et al., *A systematic review of interventions to increase breast and cervical cancer screening uptake among Asian women.* BMC Public Health, 2012. **12**: p. 413.

52. Mandelblatt, J., et al., *The cost-effectiveness of screening mammography beyond age 65 years: a systematic review for the U.S. Preventive Services Task Force.* Ann Intern Med, 2003. **139**(10): p. 835-42.

53. Mandrik O ZN, Meheus F, Severens JL, Guha N, Herrero R, Murillo R. Systematic reviews as a "lens of evidence": determinants of benefits and harms of breast cancer screening. Accepted by the International Journal of Cancer. 2019.

54. Mansfield, C., et al., *Stated Preference for Cancer Screening: A Systematic Review of the Literature, 1990-2013.* Prev Chronic Dis, 2016. **13**: p. E27.

55. Medical Advisory Secretariat. Cancer screening with digital mammography for women at average risk for breast cancer, magnetic resonance imaging (MRI) for women at high risk: an evidence-based analysis. Ont Health Technol Assess Ser [Internet]. 2010. p. 1-55

56. Moss, S.M., et al., The impact of mammographic screening on breast cancer mortality in Europe: a review of trend studies. J Med Screen, 2012. 19 Suppl 1: p. 26-32.

57. Myers, E.R., et al., Benefits and Harms of Breast Cancer Screening: A Systematic Review. Jama, 2015. 314(15): p. 1615-34.

58. Nagtegaal, I.D. and S.W. Duffy, Reduction in rate of node metastases with breast screening: consistency of association with tumor size. Breast Cancer Res Treat, 2013. 137(3): p. 653-63.

59. Nelson, H.D., et al., U.S. Preventive Services Task Force Evidence Syntheses, formerly Systematic Evidence Reviews, in Screening for Breast Cancer: Systematic Evidence Review Update for the US Preventive Services Task Force. 2009, Agency for Healthcare Research and Quality (US): Rockville (MD).

60. Nelson, H.D., et al., U.S. Preventive Services Task Force Evidence Syntheses, formerly Systematic Evidence Reviews, in Screening for Breast Cancer: A Systematic Review to Update the 2009 U.S. Preventive Services Task Force Recommendation. 2016, Agency for Healthcare Research and Quality (US): Rockville (MD).

61. Nickson, C., et al. Mammographic screening and breast cancer mortality: a case-control study and meta-analysis (Provisional abstract). Cancer Epidemiology, Biomarkers and Prevention, 2012. 21, 1479-1488.

62. Oeffinger, K.C., et al., Breast Cancer Screening for Women at Average Risk: 2015 Guideline Update From the American Cancer Society. Jama, 2015. 314(15): p. 1599-614.

63. Olsen, O. and P.C. Gotzsche, Screening for breast cancer with mammography. Cochrane Database Syst Rev, 2001(4): p. Cd001877.

64. Petticrew, M.P., et al., *False-negative results in screening programmes: systematic review of impact and implications.* Health Technol Assess, 2000. **4**(5): p. 1-120.

65. Phillips, K.A., et al., *A review of studies examining stated preferences for cancer screening.* Prev Chronic Dis, 2006. **3**(3): p. A75.

66. Puliti, D., et al., Overdiagnosis in mammographic screening for breast cancer in Europe: a literature review. J Med Screen, 2012. 19 Suppl 1: p. 42-56.

67. Rainey L, van der Waal D, Jervaeus A, Wengstrom Y, Evans DG, Donnelly LS, et al. Are we ready for the challenge of implementing risk-based breast cancer screening and primary prevention? Breast. 2018;39:24-32. Epub 2018/03/13. doi: 10.1016/j.breast.2018.02.029. PubMed PMID: 29529454.

68. Rashidian, A., et al., *Cost effectiveness of breast cancer screening using mammography; a systematic review.* Iran J Public Health, 2013. **42**(4): p. 347-57.

69. Rauscher, G.H., et al., *Accuracy of self-reported cancer-screening histories: a meta-analysis.* Cancer Epidemiol Biomarkers Prev, 2008. **17**(4): p. 748-57.

70. Ringash, J. *Preventive health care, 2001 update: screening mammography among women aged 40 - 49 years at average risk of breast cancer (Structured abstract).* Canadian Medical Association Journal, 2001. **164**, 469-476.

71. Riobo, P., et al., *[Periodic health examination].* Rev Clin Esp, 1992. **190**(7): p. 361-6.

72. Rothenberg, B.M., K.M. Ziegler, and N. Aronson, Technology evaluation center assessment synopsis: full-field digital mammography. J Am Coll Radiol, 2006. 3(8): p. 586-8.

73. Schiller-Fruhwirth, I.C., et al., Cost-Effectiveness Models in Breast Cancer Screening in the General Population: A Systematic Review. Appl Health Econ Health Policy, 2017. 15(3): p. 333-351.

74. Schmidt, A.F., et al., Differences in interaction and subgroup-specific effects were observed between randomized and nonrandomized studies in three empirical examples. J Clin Epidemiol, 2013. 66(6): p. 599-607.

75. Siedlikowski S, Ells C, Bartlett G. Scrutinizing screening: A critical interpretive review of primary care provider perspectives on mammography decisionmaking with average-risk women. Public Health Reviews. 2018;39(1). PubMed PMID: 622806131.

76. Smith, R.A., V. Cokkinides, and O.W. Brawley, *Cancer screening in the United States, 2008: A review of current American Cancer Society guidelines and cancer screening issues.* CA Cancer Journal for Clinicians, 2008. **58**(3): p. 161-179.

77. Smith, R.A., V. Cokkinides, and O.W. Brawley, *Cancer screening in the United States, 2009: A review of current American Cancer Society guidelines and issues in cancer screening.* CA Cancer Journal for Clinicians, 2009. **59**(1): p. 27-41.

78. Stone, E.G., et al., *Interventions that increase use of adult immunization and cancer screening services: a meta-analysis.* Ann Intern Med, 2002. **136**(9): p. 641-51.

79. Systematic Review of Cancer Screening Literature for Updating American Cancer Society Breast Cancer Screening Guidelines 2014, Duke Evidence Synthesis Group for American Cancer Society.

80. The benefits and harms of breast cancer screening: an independent review. Lancet, 2012. 380(9855): p. 1778-86.

81. van den Ende, C., et al., Benefits and harms of breast cancer screening with mammography in women aged 40-49 years: A systematic review. Int J Cancer, 2017. 141(7): p. 1295-1306.

82. Walter, L.C. and M.A. Schonberg, Screening mammography in older women: a review. Jama, 2014. 311(13): p. 1336-47.

83. Women's Experiences of Inaccurate Breast Cancer Screening Results: A Systematic Review and Qualitative Meta-synthesis. Ont Health Technol Assess Ser, 2016. 16(16): p. 1-22.

7. No systematic search of the literature

1. Anonymous. World Cancer Congress, WCC 2018. Journal of Global Oncology Conference: World Cancer Congress, WCC. 2018;4(Supplement 2). PubMed PMID: 627190932.
2. *The Risk for Development of Breast Cancer with the Use of Traditional Mammography Screening Practices: Clinical Evidence* 2008, CADTH
3. *Full Field Digital Mammography versus Computed Radiography for Breast Cancer Screening: A Clinical and Cost-Effectiveness Review*. 2008, CADTH.
4. *Portable and Mobile Mammography Screening Services*. 2007, CADTH.
5. *Full-field digital mammography*. 2002, Blue Cross Blue Shield Association Technology Evaluation Center. p. 1-21.
6. *National Institutes of Health Consensus Development Conference Statement: Breast Cancer Screening for Women Ages 40-49, January 21-23, 1997. National Institutes of Health Consensus Development Panel.* J Natl Cancer Inst, 1997. **89**(14): p. 1015-26.
7. *Practice Bulletin Number 42, April 2003: Breast cancer screening.* Obstetrics and Gynecology, 2003. **101**(4): p. 821-831.
8. *Mammographic screening for breast cancer: Few new data.* Prescrire International, 2008. **17**(93): p. 24-27.
9. *Mammographic breast cancer screening. Part II. Non-randomised comparisons: results similar to those of randomised trials.* Prescrire Int, 2015. **24**(159): p. 99-102.
10. Aberle, D.R., et al., *Imaging and cancer: research strategy of the American College of Radiology Imaging Network.* Radiology, 2005. **235**(3): p. 741-51.
11. Albert, U.S., et al., *2008 update of the guideline: early detection of breast cancer in Germany.* J Cancer Res Clin Oncol, 2009. **135**(3): p. 339-54.
12. Albert, U.S., et al., *Early detection of breast cancer in Germany. Guideline 2008.* Onkologe, 2008. **14**(5): p. 461-477.
13. Albert, U.S. and K.D. Schulz, *Short version of the Guideline: Early Detection of Breast Cancer in Germany. An evidence-, consensus-, and outcome-based guideline according to the German Association of the Scientific Medical Societies (AWMF) and the German Agency for Quality in Medicine (AeZQ).* J Cancer Res Clin Oncol, 2004. **130**(9): p. 527-36.
14. Albert, U.S. and K.D. Schulz, *Clinical breast examination: what can be recommended for its use to detect breast cancer in countries with limited resources?* Breast J, 2003. **9 Suppl 2**: p. S90-3.
15. Al-Foheidi, M., M.M. Al-Mansour, and E.M. Ibrahim, *Breast cancer screening: review of benefits and harms, and recommendations for developing and low-income countries.* Med Oncol, 2013. **30**(2): p. 471.
16. Alibhai, S.M.H., *Cancer screening: Applying the evidence to adults beyond age 70.* Geriatrics and Aging, 2006. **9**(3): p. 164-171.
17. Alnaimy, N.M. and N. Khoumais, *Role of Ultrasonography in Breast Cancer Imaging.* PET Clinics, 2009. **4**(3): p. 227-240.
18. Altobelli, E., et al., Breast Cancer Screening Programmes across the WHO European Region: Differences among Countries Based on National Income Level. Int J Environ Res Public Health, 2017. 14(4).

19. Alzaghal AA, DiPiro P. Applications of Advanced Breast Imaging Modalities. Curr Oncol Rep. 2018 May 29;20(7):57. doi: 10.1007/s11912-018-0700-3.

20. Arleo, E.K., et al., *Screening mammography for women in their 40s: A retrospective study of the potential impact of the U.S. preventive service task force's 2009 breast cancer screening recommendations.* American Journal of Roentgenology, 2013. **201**(6): p. 1401-1406.

21. Arroyave, A.M., E.K. Penaranda, and C.L. Lewis Organizational change: a way to increase colon, breast and cervical cancer screening in primary care practices (Structured abstract). Journal of Community Health, 2011. 36, 281-288.

22. Alsheh Ali M, Eriksson M, Czene K, Hall P, Humphreys K. Detection of potential microcalcification clusters using multivendor for-presentation digital mammograms for short-term breast cancer risk estimation. Medical Physics. 2019;46(4):1938-46. PubMed PMID: 626726712.

23. Baines, C.J., *Rational and irrational issues in breast cancer screening.* Cancers, 2011. **3**(1): p. 252-266.

24. Baines, C.J., *Frank words about breast screening.* Open Med, 2011. **5**(3): p. e134-6.

25. Baker, S., M. Wall, and A. Bloomfield, Breast cancer screening for women aged 40 to 49 years--what does the evidence mean for New Zealand? N Z Med J, 2005. 118(1221): p. U1628.

26. Baltzer PAT, Schulz-Wendtland R. Future developments in breast imaging. [German]. Gynakologe. 2018;51(5):362-9.

27. Baron, R.C., et al., *Intervention to increase recommendation and delivery of screening for breast, cervical, and colorectal cancers by healthcare providers a systematic review of provider reminders.* Am J Prev Med, 2010. **38**(1): p. 110-7.

28. Baron, R.C., et al., *Client-directed interventions to increase community demand for breast, cervical, and colorectal cancer screening a systematic review.* Am J Prev Med, 2008. **35**(1 Suppl): p. S34-55.

29. Barton H, Shatti D, Jones CA, Sakthithasan M, Loughborough WW. Review of radiological screening programmes for breast, lung and pancreatic malignancy. Quantitative Imaging in Medicine and Surgery. 2018;8(5):525-34. PubMed PMID: 623797550.

30. Beral, V., et al., *The number of women who would need to be screened regularly by mammography to prevent one death from breast cancer.* J Med Screen, 2011. **18**(4): p. 210-2.

31. Berg, W.A., *Tailored supplemental screening for breast cancer: what now and what next?* AJR Am J Roentgenol, 2009. **192**(2): p. 390-9.

32. Berg, W.A. and E.B. Mendelson, *How should screening breast US be audited? The patient perspective.* Radiology, 2014. **272**(2): p. 309-315.

33. Berry, D.A., *Benefits and risks of screening mammography for women in their forties: a statistical appraisal.* J Natl Cancer Inst, 1998. **90**(19): p. 1431-9.

34. Berry, D.A., et al., Effect of screening and adjuvant therapy on mortality from breast cancer. N Engl J Med, 2005. 353(17): p. 1784-92.

35. Black E, Richmond R. Improving early detection of breast cancer in sub-Saharan Africa: Why mammography may not be the way forward. Globalization and Health. 2019;15(1). PubMed PMID: 625827261.

36. Boer, R., S. Plevritis, and L. Clarke, *Diversity of model approaches for breast cancer screening: a review of model assumptions by the Cancer Intervention and Surveillance Network (CISNET) Breast Cancer Groups.* Stat Methods Med Res, 2004. **13**(6): p. 525-38.

37. Boyle, P., *Current situation of screening for cancer.* Ann Oncol, 2002. **13 Suppl 4**: p. 189-98.

38. Brem, R.F., *The Never-Ending Controversies of Screening Mammography: What Is the Appropriate Callback Rate for Women Undergoing Screening Mammographic Examination?* Cancer, 2004. **100**(8): p. 1549-1552.

39. Brodersen, J., K.J. Jorgensen, and P.C. Gotzsche, *The benefits and harms of screening for cancer with a focus on breast screening.* Pol Arch Med Wewn, 2010. **120**(3): p. 89-94.

40. Brouwers, M.C., et al., *Effective interventions to facilitate the uptake of breast, cervical and colorectal cancer screening: an implementation guideline.* Implement Sci, 2011. **6**: p. 112.

41. Bryan, T. and E. Snyder, *The clinical breast exam: A skill that should not be abandoned.* Journal of General Internal Medicine, 2013. **28**(5): p. 719-722.

42. Bryant, H. and V. Mai, *Impact of age-specific recommendation changes on organized breast screening programs.* Prev Med, 2011. **53**(3): p. 141-3.

43. Burhenne, L.J.W. and H.J. Burhenne, *The Canadian National Breast Screening Study: A Canadian critique.* American Journal of Roentgenology, 1993. **161**(4): p. 761-763.

44. Burrell, H.C. and A.J. Evans, *Radiological assessment of the breast: what the Surgical Oncologist needs to know.* Eur J Surg Oncol, 2001. **27**(7): p. 689-91.

45. Calonge, N., et al., *Screening for breast cancer: U.S. preventive services task force recommendation statement.* Annals of Internal Medicine, 2009. **151**(10): p. 716-726.

46. Caverly, T.J., et al., *Presentation of Benefits and Harms in US Cancer Screening and Prevention Guidelines: Systematic Review.* J Natl Cancer Inst, 2016. **108**(6): p. djv436.

47. Champion, V.L., S.M. Rawl, and U. Menon, *Population-based cancer screening.* Oncol Nurs Forum, 2002. **29**(5): p. 853-61.

48. Chiarelli, A.M., et al., *Favourable prognostic factors of subsequent screen-detected breast cancers among women aged 50-69.* Eur J Cancer Prev, 2012. **21**(6): p. 499-506.

49. Chiolero, A., et al., *How to prevent overdiagnosis.* Swiss Medical Weekly, 2015. **145**.

50. Ciatto, S., *Controversies regarding screening mammography.* Crit Rev Oncol Hematol, 1997. **26**(1): p. 55-9.

51. Clarke, L.D., et al., *A comparative review of CISNET breast models used to analyze U.S. breast cancer incidence and mortality trends.* J Natl Cancer Inst Monogr, 2006(36): p. 96-105.

52. Coleman, C., Early Detection and Screening for Breast Cancer. Semin Oncol Nurs, 2017. 33(2): p. 141-155.

53. Collarino A, Fuoco V, Arias-Bouda LMP, Sanchez AM, de Geus-Oei LF, Masetti R, et al. Novel frontiers of dedicated molecular imaging in breast cancer diagnosis. Translational Cancer Research. 2018;7(Supplement3):S295-S306. PubMed PMID: 621874264.

54. Cook TS. Large datasets, logistics, sharing and workflow in screening. British Journal of Radiology. 2018;91(1090). PubMed PMID: 624320722.

55. Corbex, M., R. Burton, and H. Sancho-Garnier, *Breast cancer early detection methods for low and middle income countries, a review of the evidence.* Breast, 2012. **21**(4): p. 428-434.

56. Cox, B., *Variation in the effectiveness of breast screening by year of follow-up.* J Natl Cancer Inst Monogr, 1997(22): p. 69-72.

57. Dejean, D., et al., *Women's experiences of inaccurate breast cancer screening results: A systematic review and qualitative meta-synthesis.* Ontario Health Technology Assessment Series, 2016. **16**(16): p. 1-22.

58. Demment, M.M., et al., *Developing the Evidence Base to Inform Best Practice: A Scoping Study of Breast and Cervical Cancer Reviews in Low- and Middle-Income Countries.* PLoS One, 2015. **10**(9): p. e0134618.

59. Dodd, G.D., *American Cancer Society guidelines on screening for breast cancer: An overview.* Ca-A Cancer Journal for Clinicians, 1992. **42**(3): p. 177-180.

60. Doede AL, Mitchell EM, Wilson D, Panagides R, Oria MOB. Knowledge, beliefs, and attitudes about breast cancer screening in Latin America and the Caribbean: An in-depth narrative review. Journal of Global Oncology. 2018;(pagination). PubMed PMID: 624435397.

61. Doke K, Butler S, Mitchell MP. Current Therapeutic Approaches to DCIS. Journal of Mammary Gland Biology and Neoplasia. 2018;23(4):279-91. PubMed PMID: 624182359.

62. Donnelly, T.T., et al., Arab women's breast cancer screening practices: a literature review. Asian Pac J Cancer Prev, 2013. 14(8): p. 4519-28.

63. Duffy, S.W., et al., *Rapid review of evaluation of interventions to improve participation in cancer screening services.* J Med Screen, 2017. **24**(3): p. 127-145.

64. El Saghir, N.S. and R.N. Charara, *International screening and early detection of breast cancer: Resource-sensitive, age- and risk-specific guidelines.* Breast Cancer Management, 2014. **3**(5): p. 397-407.

65. Esserman, L. and K. Kerlikowske, *Should we recommend screening mammography for women aged 40 to 49?* Oncology (Williston Park), 1996. **10**(3): p. 357-64; discussion: 370-6.

66. Euhus, D., P.A. Di Carlo, and N.F. Khouri, *Breast Cancer Screening.* Surgical Clinics of North America, 2015. **95**(5): p. 991-1011.

67. Feig, S.A., *Mammographic screening of women aged 40 to 49 years. Is it justified?* Obstet Gynecol Clin North Am, 1994. **21**(4): p. 587-606.

68. Feig, S.A., *Screening mammography: A successful public health initiative.* Revista Panamericana de Salud Publica/Pan American Journal of Public Health, 2006. **20**(2-3): p. 125-133.

69. Ferrigni E, Bergom C, Yin Z, Szabo A, Kong AL. Breast Cancer in Women Aged 80 Years or Older: An Analysis of Treatment Patterns and Disease Outcomes. Clin Breast Cancer. 2019 Jun;19(3):157-164.

70. Fletcher, S.W., *Breast cancer screening among women in their forties: an overview of the issues.* J Natl Cancer Inst Monogr, 1997(22): p. 5-9.

71. Fletcher, S.W., *Breast cancer screening: A 35-year perspective.* Epidemiologic Reviews, 2011. **33**(1): p. 165-175.

72. Fletcher, S.W., et al., *Report of the International Workshop on Screening for Breast Cancer.* Journal of the National Cancer Institute, 1993. **85**(20): p. 1644-1656.

73. Fletcher, S.W. and J.G. Elmore, *Clinical practice. Mammographic screening for breast cancer.* N Engl J Med, 2003. **348**(17): p. 1672-80.

74. Fuller, M.S., C.I. Lee, and J.G. Elmore, *Breast cancer screening: an evidence-based update.* Med Clin North Am, 2015. **99**(3): p. 451-68.

75. Gelband, H., et al., *Costs, affordability, and feasibility of an essential package of cancer control interventions in low-income and middle-income countries: key messages from Disease Control Priorities, 3rd edition.* Lancet, 2016. **387**(10033): p. 2133-2144.

76. Gennari, R., et al., *Early detection of cancer: ideas for a debate.* Crit Rev Oncol Hematol, 2007. **61**(2): p. 97-103.

77. Glasziou, P. and L. Irwig, *The quality and interpretation of mammographic screening trials for women ages 40-49.* J Natl Cancer Inst Monogr, 1997(22): p. 73-7.

78. Gotzsche, P.C., et al., *Why mammography screening has not lived up to expectations from the randomised trials.* Cancer Causes Control, 2012. **23**(1): p. 15-21.

79. Hall, F.M., *Screening mammography guidelines: an alternative proactive approach.* Radiology, 2014. **273**(3): p. 646-51.

80. Harris, J.R., et al., *Breast cancer (1).* N Engl J Med, 1992. **327**(5): p. 319-28.

81. Harris, J.R., et al., *Breast cancer (2).* N Engl J Med, 1992. **327**(6): p. 390-8.

82. Harris, J.R., et al., *Breast cancer (3).* N Engl J Med, 1992. **327**(7): p. 473-80.

83. Hendrick, R.E., et al., *Benefit of screening mammography in women aged 40-49: a new meta-analysis of randomized controlled trials.* J Natl Cancer Inst Monogr, 1997(22): p. 87-92.

84. Heywang-Koebrunner, S., et al., *Mammography screening - As of 2013.* Geburtshilfe und Frauenheilkunde, 2013. **73**(10): p. 1007-1016.

85. Hofvind, S., et al., *False-positive results in mammographic screening for breast cancer in Europe: a literature review and survey of service screening programmes.* J Med Screen, 2012. **19 Suppl 1**: p. 57-66.

86. Hsairi M, Mehdi F, Bellaaj R, Kassis M. Health screening strategies in maghreb countries: Situation analysis and perspectives. [French]. Tunisie Medicale. 2018;96(10-11):688-95. PubMed PMID: 2001588217.

87. Ismail H.M., Pretty C.G., Signal M.K., Haggers M., Chase J.G. Attributes, performance, and gaps in current & emerging breast cancer screening technologies. Current Medical Imaging Reviews, 2019. 15(2): 122-131.

88. Jacklyn, G., et al., *Meta-analysis of breast cancer mortality benefit and overdiagnosis adjusted for adherence: improving information on the effects of attending screening mammography.* Br J Cancer, 2016. **114**(11): p. 1269-76.

89. Jafari SH, Saadatpour Z, Salmaninejad A, et al. Breast cancer diagnosis: Imaging techniques and biochemical markers. J Cell Physiol. 2018 Jul;233(7):5200-5213.

90. Jatoi, I., *Breast cancer screening.* American Journal of Surgery, 1999. **177**(6): p. 518-524.

91. Kale MS, Korenstein D. Overdiagnosis in primary care: Framing the problem and finding solutions. Bmj. 2018;362(no pagination). PubMed PMID: 623474111.

92. Katzen J1, Dodelzon K2. A review of computer aided detection in mammography. Clin Imaging. 2018 Nov - Dec;52:305-309.

93. Kerlikowske, K., *Efficacy of screening mammography among women aged 40 to 49 years and 50 to 69 years: comparison of relative and absolute benefit.* J Natl Cancer Inst Monogr, 1997(22): p. 79-86.

94. Koleva-Kolarova, R.G., et al., *To screen or not to screen for breast cancer? How do modelling studies answer the question?* Curr Oncol, 2015. **22**(5): p. e380-2.

95. de Koning, H.J., *Mammographic screening: evidence from randomised controlled trials.* Annals of Oncology, 2003. **14**(8): p. 1185-1189.

96. Kopans DB. Breast cancer screening: Where have we been and where are we going? A personal perspective based on history, data and experience. Clin Imaging. 2018 Mar - Apr;48:vii-xi. doi: 10.1016/j.clinimag.2017.12.016.

97. Kopans, D.B., *The 2009 US Preventive Services Task Force (USPSTF) guidelines are not supported by science: the scientific support for mammography screening.* Radiol Clin North Am, 2010. **48**(5): p. 843-57.

98. Lauby-Secretan, B., et al., *Breast-cancer screening--viewpoint of the IARC Working Group.* N Engl J Med, 2015. **372**(24): p. 2353-8.

99. Lee KA, Talati N, Oudsema R, Steinberger S, Margolies LR. BI-RADS 3: Current and Future Use of Probably Benign. Curr Radiol Rep. 2018;6(2):5.

100. Levinsohn E, Altman M, Chagpar AB. Controversies regarding the diagnosis and management of ductal carcinoma in situ. American Surgeon. 2018;84(1):1-6. PubMed PMID: 620694557.

101. Lima ZS, Ebadi MR2 Amjad G, Younesi L. Application of Imaging Technologies in Breast Cancer Detection: A Review Article. Open Access Maced J Med Sci. 2019 Mar 14;7(5):838-848. doi: 10.3889/oamjms.2019.171. eCollection 2019 Mar 15.

102. Loberg, M., et al., *Benefits and harms of mammography screening.* Breast Cancer Res, 2015. **17**: p. 63.

103. Lowes S, Leaver A, Redman A. Diagnostic and interventional imaging techniques in breast cancer. Surgery (United Kingdom). 2019;37(3):140-50. PubMed PMID: 2001610102.

104. Mango V, Bryce Y, Morris EA, Gianotti E, Pinker K. Commentary acog practice Bulletin July 2017: Breast cancer risk assessment and screening in average-risk Women. British Journal of Radiology. 2018;91(1090).

105. Marmot, M.G., et al., *The benefits and harms of breast cancer screening: an independent review.* Br J Cancer, 2013. **108**(11): p. 2205-40.

106. McDonald, S., D. Saslow, and M.H. Alciati, *Performance and reporting of clinical breast examination: a review of the literature.* CA Cancer J Clin, 2004. **54**(6): p. 345-61.

107. McCready, T., D. Littlewood, and J. Jenkinson, Breast self-examination and breast awareness: a literature review. J Clin Nurs, 2005. 14(5): p. 570-8.

108. Meuwly, J.Y., *[Ultrasound for Breast Cancer Screening: an Effective Tool in a Personalized Screening].* Praxis (Bern 1994), 2015. **104**(25): p. 1399-404.

109. Mushlin, A.I., R.W. Kouides, and D.E. Shapiro, *Estimating the accuracy of screening mammography: A meta-analysis.* American Journal of Preventive Medicine, 1998. **14**(2): p. 143-153.

110. Nazari SS, Mukherjee P. An overview of mammographic density and its association with breast cancer. Breast Cancer. 2018 May;25(3):259-267.

111. Narayanan D, Berg WA. Use of Breast-Specific PET Scanners and Comparison with MR Imaging. Magnetic Resonance Imaging Clinics of North America. 2018;26(2):265-72. PubMed PMID: 2000621820.Nystrom, L., et al., *Breast cancer screening with mammography: overview of Swedish randomised trials.* Lancet, 1993. **341**(8851): p. 973-8.

112. Perry, N., et al., *European guidelines for quality assurance in breast cancer screening and diagnosis. Fourth edition--summary document.* Ann Oncol, 2008. **19**(4): p. 614-22.

113. Petitti, D.B., et al., *Breast cancer screening: From science to recommendation.* Radiology, 2010. **256**(1): p. 8-14.

114. Ravert, P.K. and C. Huffaker, *Breast cancer screening in women: An integrative literature review.* J Am Acad Nurse Pract, 2010. **22**(12): p. 668-73.

115. Riogi, B., Wasike, R. Breast cancer at a glance. Annals of African Surgery, 2018. 15(3): 1-3.

116. Sabatino, S.A., et al., Interventions to Increase Recommendation and Delivery of Screening for Breast, Cervical, and Colorectal Cancers by Healthcare Providers. Systematic Reviews of Provider Assessment and Feedback and Provider Incentives. American Journal of Preventive Medicine, 2008. 35(1 SUPPL.): p. S67-S74.

117. Schopper, D. and C. de Wolf, *How effective are breast cancer screening programmes by mammography? Review of the current evidence.* Eur J Cancer, 2009. **45**(11): p. 1916-23.

118. Seely JM, Alhassan T. Screening for breast cancer in 2018-what should we be doing today? Current Oncology. 2018;25(Supplement 1):S115-S24. PubMed PMID: 622658552.

119. Shaheed SU, Tait C, Kyriacou K, Linforth R, Salhab M, Sutton C. Evaluation of nipple aspirate fluid as a diagnostic tool for early detection of breast cancer. Clinical Proteomics. 2018;15(1). PubMed PMID: 620162027.

120. Shapiro, S., *The status of breast cancer screening: a quarter of a century of research.* World J Surg, 1989. **13**(1): p. 9-18.

121. Sickles, E.A. and D.B. Kopans, *Mammographic screening for women aged 40 to 49 years: the primary care practitioner's dilemma.* Ann Intern Med, 1995. **122**(7): p. 534-8.

122. Sinha VC, Piwnica-Worms H. Intratumoral Heterogeneity in Ductal Carcinoma In Situ: Chaos and Consequence. Journal of Mammary Gland Biology and Neoplasia. 2018;23(4):191-205. PubMed PMID: 623950361.

123. Sitt JCM, Lui CY, Sinn LHY, Fong JCY. Understanding breast cancer screening-past, present, and future. Hong Kong Medical Journal. 2018;24(2):166-74.

124. Smith, R.A., et al., *Cancer screening in the United States, 2016: A review of current American Cancer Society guidelines and current issues in cancer screening.* CA Cancer J Clin, 2016. **66**(2): p. 96-114.

125. Smith, R.A., et al., *Cancer screening in the United States, 2015: A review of current American Cancer Society guidelines and current issues in cancer screening.* CA Cancer Journal for Clinicians, 2015. **65**(1): p. 30-54.

126. Smith, R.A., et al., *Cancer screening in the United States, 2014: A review of current American Cancer Society guidelines and current issues in cancer screening.* CA Cancer Journal for Clinicians, 2014. 64(1): p. 30-51.

127. Smith, R.A., V. Cokkinides, and O.W. Brawley, *Cancer screening in the United States, 2012: A review of current American Cancer Society guidelines and current issues in cancer screening.* CA Cancer Journal for Clinicians, 2012. 62(2): p. 129-142.

128. Smith, R.A., S.W. Duffy, and L. Tabar, *Breast cancer screening: the evolving evidence.* Oncology (Williston Park), 2012. 26(5): p. 471-5, 479-81, 485-6.

129. Smith, R.A., et al., *Cancer screening in the United States, 2010: a review of current American Cancer Society guidelines and issues in cancer screening.* CA Cancer J Clin, 2010. 60(2): p. 99-119.

130. Smith, R.A., V. Cokkinides, and H.J. Eyre, *American Cancer Society guidelines for the early detection of cancer, 2004.* CA Cancer J Clin, 2004. 54(1): p. 41-52.

131. Smith, R.A., et al., *The randomized trials of breast cancer screening: what have we learned?* Radiol Clin North Am, 2004. 42(5): p. 793-806, v.

132. Smith, R.A., V. Cokkinides, and H.J. Eyre, *American Cancer Society guidelines for the early detection of cancer, 2003.* Ca-A Cancer Journal for Clinicians, 2003. 53(1): p. 27-43.

133. Smith, R.A., et al., *American Cancer Society Guidelines for breast cancer screening: Update 2003.* Ca-A Cancer Journal for Clinicians, 2003. 53(3): p. 141-169.

134. Stomper, P.C. and R.S. Gelman, *Mammography in symptomatic and asymptomatic patients.* Hematol Oncol Clin North Am, 1989. 3(4): p. 611-40.

135. Suter MB, Pagani O. Should age impact breast cancer management in young women? Fine tuning of treatment guidelines. Therapeutic Advances in Medical Oncology. 2018;10(no pagination). PubMed PMID: 624227851.

136. Suzuki, A., T. Ishida, and N. Ohuchi, *Controversies in breast cancer screening for women aged 40-49 years.* Jpn J Clin Oncol, 2014. 44(7): p. 613-8.

137. Tamaki, K., et al., *The challenge to reduce breast cancer mortality in Okinawa: consensus of the first Okinawa breast oncology meeting.* Jpn J Clin Oncol, 2013. 43(2): p. 208-13.

138. Thigpen, D., A. Kappler, and R. Brem, The Role of Ultrasound in Screening Dense Breasts-A Review of the Literature and Practical Solutions for Implementation. Diagnostics (Basel), 2018. 8(1).

139. Virgini, V., et al., *Check-up examination: Recommendations in adults.* Swiss Medical Weekly, 2015. 145.

140. Vourtsis A, Berg WA. Breast density implications and supplemental screening. European Radiology. 2019;29(4):1762-77. PubMed PMID: 624078951.

141. Warner, E., R. Heisey, and J.C. Carroll, *Applying the 2011 Canadian guidelines for breast cancer screening in practice.* Cmaj, 2012. 184(16): p. 1803-7.

142. Willems B, Bracke P. Participants, Physicians or Programmes: Participants' educational level and initiative in cancer screening. Health Policy. 2018;122(4):422-30. Epub 2018/02/20. doi: 10.1016/j.healthpol.2018.02.001. PubMed PMID: 29454541.

143. Woolf, S.H., *The accuracy and effectiveness of routine population screening with mammography, prostate-specific antigen, and prenatal ultrasound: a review of published scientific evidence.* Int J Technol Assess Health Care, 2001. 17(3): p. 275-304.

144. Yabroff, K.R. and J.S. Mandelblatt, *Interventions targeted toward patients to increase mammography use.* Cancer Epidemiol Biomarkers Prev, 1999. 8(9): p. 749-57.

145. Zendehdel M, Niakan B, Keshtkar A, Rafiei E, Salamat F. Subtypes of benign breast disease as a risk factor for breast cancer: A systematic review and meta-analysis protocol. Iranian Journal of Medical Sciences. 2018;43(1). PubMed PMID: 620024405.

146. Zonderland, H.M., *The role of ultrasound in the diagnosis of breast cancer.* Semin Ultrasound CT MR, 2000. 21(4): p. 317-24.

8. No individual evaluation

1. *Ontario health technology assessment series ultrasound as an adjunct to mammography for breast cancer screening: A health technology assessment.* Ontario Health Technology Assessment Series, 2016. **16**(15): p. 1-71.
2. *Recommendations on screening for breast cancer in average-risk women aged 40-74 years.* CMAJ, 2011. **183**(17): p. 1991-2001.
3. Ackerson, K. and S.D. Preston, *A decision theory perspective on why women do or do not decide to have cancer screening: systematic review.* J Adv Nurs, 2009. **65**(6): p. 1130-40.
4. Baker, S., M. Wall, and A. Bloomfield, *What is the most appropriate breast-cancer screening interval for women aged 45 to 49 years in New Zealand?* N Z Med J, 2005. **118**(1221): p. U1636.
5. Pace, L.E. and N.L. Keating, *A systematic assessment of benefits and risks to guide breast cancer screening decisions.* Jama, 2014. **311**(13): p. 1327-35.

9. Duplicates

1. Albert, U.S., et al., *[Summary of the updated stage 3 guideline for early detection of breast cancer in Germany 2008].* Rofo, 2008. **180**(5): p. 455-65.
2. Gartlehner, G., et al. *Adjunct ultrasonography for breast cancer screening in women at average risk: a systematic review (Structured abstract)*. International Journal of Evidence-Based Healthcare, 2013. **11**, 87-93.
3. Gotzsche, P.C. and M. Nielsen, *Screening for breast cancer with mammography.* Cochrane Database Syst Rev, 2011(1): p. Cd001877.
4. Hamashima, C., et al., *A meta-analysis of mammographic screening with and without clinical breast examination.* Cancer Sci, 2015. **106**(7): p. 812-8.
5. Nelson, H.D., et al. *Screening for breast cancer: an update for the U.S. preventive services task force (Structured abstract)*. Annals of Internal Medicine, 2009. **151**, 727-737.
6. Nelson, H.D., et al., *Effectiveness of Breast Cancer Screening: Systematic Review and Meta-analysis to Update the 2009 U.S. Preventive Services Task Force Recommendation.* Ann Intern Med, 2016. **164**(4): p. 244-55.
7. Nelson, H.D., et al., *Harms of Breast Cancer Screening: Systematic Review to Update the 2009 U.S. Preventive Services Task Force Recommendation.* Ann Intern Med, 2016. **164**(4): p. 256-67.

10. Not in English

1. Bozorgi N, Khani S, Elyasi F, Moosazadeh M, Janbabaei G, Shojaee L. A review of strategies to promote breast cancer screening behaviors in women. Journal of Mazandaran University of Medical Sciences. 2018;28(165):243-55.

Appendix C. Reviews reporting on uptake of breast cancer screening

Author (first) year	Search: (a) Years (b) Region searched/ Targeted	Funding source indicated	Inclusion: (a) Technology/comparators (b) Type of studies included	Analysis: (a) Meta-analysis (b) Methods for quality assessment	Key Review topic	(a) Countries included (b) Applicability*	AMSTAR score[1]
**Reviews focusing on interventions to improve participation rate**							
Wagner, 1998 [1]	(a) 1985-1996<br>(b) world/ the USA	National Institutes on Aging Predoctoral Traineeship, GK09 405940 31028	(a) Mailed BCS reminders vs controls<br>(b) RCTs	(a) Yes<br>(b) None reported	(1) Effectiveness; (2) costs; (3) areas in need of further research of mailed patient reminders	(a) The USA, Australia, the NZ<br>(b) HIC	2
Sin, 1999 [2]	(a) 1980-1998<br>(b) generalizable to the UK /UK	None	(a) Interventions to increase participation in MM<br>(b) No limitation to study design	(a) No<br>(b) By the principles of the USA and Canadian Task force	The effectiveness of the different interventions to increase BCS uptake	(a) The UK, Australia, the USA<br>(b) HIC	5
Jepson, 2000 [3]	(a) 1998<br>(b) world/ the UK	National Health Service	(a) Screenings/ technologies promoting screening<br>(b) RCTs, controlled trials, cohort studies or case–control studies	(a) Yes<br>(b) Criteria based on validity checklists in CRD Report Number 4 (response rate, blinding etc.)	Factors associated with the uptake of screening programmes and the effectiveness of methods used to increase uptake	(a) The USA (29 of 32 studies on MM), the UK, Australia, Italy<br>(b) HIC	6
Bonfill, 2001 [4]	(a) 1966-2000<br>(b) world	None	(a) Interventions to increase participation in MM vs no active intervention<br>(b) RCT or controlled trials	(a) Yes<br>(b) No standard instrument; the assessment is based on randomization	Effectiveness of different strategies for increasing the participation rate of women invited to community BCS activities or MM programs	(a) The USA, the UK, Australia<br>(b) HIC	7

Author (first) year	Search: (a) Years (b) Region searched/ Targeted	Funding source indicated	Inclusion: (a) Technology/comparators (b) Type of studies included	Analysis: (a) Meta-analysis (b) Methods for quality assessment	Key Review topic	(a) Countries included (b) Applicability*	AMSTAR score[1]
Ratner, 2001 [5]	(a) 1966-1997<br>(b) world	None	(a) Technologies promoting MM screening<br>(b) RCTs, controlled trials, cohort studies or case–control studies	(a) Yes<br>(b) Randomization, pre-test screening status, and information verification	Factors that influence effectiveness of interventions in increasing women's use of MM screening programs	(a) Not clear, >80% in the USA<br>(b) Unclear	2
Denhaerynck, 2003 [6]	(a) 1981-2001<br>(b) world	European Commission (Europe Against Cancer Program)	(a) Direct-contact strategies on breast cancer–screening<br>(b) (Quasi-)RCT	(a) Yes<br>(b) Risk of bias, no instrument is reported	The effectiveness of direct-contact strategies on BCS attendance	(a) The USA, Europe, Australia<br>(b) HIC	7
O'Malley, 2003 [7]	(a) 1985-2003<br>(b) world (with Hispanic population >5%)	National Cancer Institute	(a) Interventions used to control cancer prevention<br>(b) RCT, quasi-experimental, or pre–post design controlled trial	(a) No<br>(b) None reported, though stated that it was assessed	To examine published literature describing primary care–based cancer control interventions for Latinos and to identify avenues for future interventions	(a) The USA<br>(b) The USA	4
Sohl, 2007 [8]	(a) 1997 - 2005<br>(b) world	Stony Brook University / the National Cancer Institute	(a) Intervention aimed to increase use of MM screening<br>(b) Experimental or quasiexperimental design	(a) Yes<br>(b) Not stated	The effectiveness of tailored interventions, designed to reach one specific person based on her unique characteristics, for promoting MM use.	(a) Unclear<br>(b) Unclear	4

Author (first) year	Search: (a) Years (b) Region searched/ Targeted	Funding source indicated	Inclusion: (a) Technology/comparators (b) Type of studies included	Analysis: (a) Meta-analysis (b) Methods for quality assessment	Key Review topic	(a) Countries included (b) Applicability*	AMSTAR score[1]
Vernon, 2010 [9]	(a) 1966-2009<br>(b) world	National Cancer Institute; American Cancer Society Grant	(a) Interventions to promote repetitive MM vs no interventions<br>(b) Intervention studies	(a) Yes<br>(b) Completeness of reporting on selected aspects of internal and external validity	Efficacy of interventions designed to promote regular MM screening	(a) All except one the USA (not clearly reported)<br>(b) USA	8
Brouwers, 2011 [10]	(a) Jepson review as a background; 2004-2008; update 2010<br>(b) world/ Canada	Ontario Ministry of Health through Cancer Care Ontario	(a) Client reminders, client incentives, mass media, small media, group education, one-on-one education, reducing structural barriers, reducing out-of-pocket costs, provider audit feedback and provider incentives<br>(b) RCTs or cluster RCTs	(a) Yes<br>(b) The RCTs and cluster RCTs were evaluated along eight criteria: funding, randomization method, baseline characteristics, blinding, statistical power, achievement of target sample size, follow-up, and intention-to-treat analysis	What interventions have been shown to increase the uptake of cancer screening by individuals, specifically for breast, cervical, and colorectal cancer screening?	(a) The USA, Canada<br>(b) North America	4
Edwards, 2013 [11]	(a) 1985-2005<br>(b) world	Welsh Assembly Government, UK	(a) Personalised and general risk communication interventions<br>(b) RCTs	(a) Yes<br>(b) Risk of bias (selection, performance, detection, attrition, reporting)	The effects of personalised and general risk communication interventions in promoting an informed decision about participating in health screening and the effects of these	(a) The USA<br>(b) The USA	9

Author (first) year	Search: (a) Years (b) Region searched/ Targeted	Funding source indicated	Inclusion: (a) Technology/comparators (b) Type of studies included	Analysis: (a) Meta-analysis (b) Methods for quality assessment	Key Review topic	(a) Countries included (b) Applicability*	AMSTAR score[1]
					interventions on people's cognitive, affective and behavioural outcomes.		
Bellhouse, 2017 [12]	(a) 2017<br>(b) world	None	(a) Community-based health worker (CBHW) interventions<br>(b) RCT- and cluster-RCTs	(a) Yes<br>(b) Risk of bias.	Effectiveness of CBHWs in promoting outcomes pertinent to early cancer diagnosis including screening, symptom recognition, early detection, and help-seeking behaviour	(a) The USA (all except one)<br>(b) The USA	4
**Cluster articles Camilloni and Ferroni (2012-2013)**							
Ferroni, 2012 [31]	(a) Jepson et al. review (2000).; 1999- 2009<br>(b) world /Italy	the Ministry of Health of Italy	(a) breast, cervical, and colon cancer screening invitations (by spontaneous screening; invitation by letter, invitation by GP);<br>(b) RCT, experimental studies, and before-and-after studies	(a) Yes<br>(b) CONSORT (for RCT) and STROBE checklist (for cohort or cross-sectional studies), then CASP criteria	Effectiveness of two different types of screening programs – by letter invitation and GP-based – on screening uptake for breast, cervical and colorectal cancers compared to spontaneous testing	(a) The USA, the UK, Australia;<br>(b) HIC	4

Author (first) year	Search: (a) Years (b) Region searched/ Targeted	Funding source indicated	Inclusion: (a) Technology/comparators (b) Type of studies included	Analysis: (a) Meta-analysis (b) Methods for quality assessment	Key Review topic	(a) Countries included (b) Applicability*	AMSTAR score[1]
Camilloni, 2013[32]	(a) 1999-2012 (b) world /Italy	Ministry of Health of Italy	(a) Interventions to increase participation in organized cervical, breast, and colorectal screenings vs standard postal invitation letter (b) Experimental studies	(a) Yes (b) Risk of bias (CONSORT list, CASP criteria and the Cochrane Collaboration tool). Observational or almost-experimental studies assessed with the STROBE checklist and then with CASP criteria	Interventions to increase participation in organised cervical, breast, and colorectal screening programmes, using the standard invitation letter as comparator for all the proposed interventions	(a) The USA, Australia, the NZ, Germany, the UK, Italy, Spain, Chile, Singapore. (b) HIC	7
**Reviews focused on behaviour of screened population and associated factors**							
Curbow, 2004 [13]	(a) 2003 (b) world	None	(a) Community-based interventions (b) Experimental or quazi-experimental	(a) No (b) None stated	Identify behaviours in older adults that could be targeted to reduce the risk of disease and to promote healthy aging and to identify effective strategies for doing so.	(a) The USA, Singapore (b) HIC	3
Soler-Michel, 2005 [14]	(a) 1990 - 2003 (b) world/ France	None	(a) Mass screening (b) prospective and retrospective observational studies	(a) No (b) Not stated	Secondary participation factors for breast cancer screening	(a) The UK, the NL, Sweden, NZ, Canada, Australia,	3

Author (first) year	Search: (a) Years (b) Region searched/ Targeted	Funding source indicated	Inclusion: (a) Technology/comparators (b) Type of studies included	Analysis: (a) Meta-analysis (b) Methods for quality assessment	Key Review topic	(a) Countries included (b) Applicability*	AMSTAR score[1]
						Spain, Belgium<br>(b) HIC	
Brewer, 2007 [15]	a) 2006<br>(b) world/the USA	The University of North Carolina Lineberger Comprehensive Cancer Center and the American Cancer Society	a) BCS vs no screening<br>(b) Not clear; observational included.	(a) Yes<br>(b) Studies were excluded based on quality criteria. No reporting on quality	The long-term effects of false-positive screening MM on the behaviour and well-being of women 40 years of age or older	(a) Europe, the USA, Canada, Australia<br>(b) HIC	5
Autier, 2009 [16]	Search from another reviews	None	(a) MM vs no screening<br>(b) RCTs and other reviews as a source of RCT	(a) Yes<br>(b) Not clear, simply stated that the quality was assessed	How changes in the risk of being diagnosed with advanced BC are associated with changes in death from BC, taking into account all the available data from the RCT on MM screening	(a) the USA, Canada, the UK, Sweden<br>(b) HIC	1
Asadzadeh, 2011 [17]	(a) 2005-2011<br>(b) Limited resource countries/ Iran	Not specified	(a) Not specified<br>(b) Not specified	(a) No<br>(b) Not stated	Available literature and considerations for launching a successful mass screening program in limited resource countries	(a) Iran, Nigeria, Turkey, Pakistan, India<br>(b) Unclear	1
Donnelly, 2013 [18]	(a) 2000-2011<br>(b) Arab countries	Qatar National Research Fund under its National Priority Research Program (NPRP 09-261-3-059)	(a) BCS practices in the Arab world<br>(b) not specified	(a) No<br>(b) Not stated	1) Gain an overview of BCS practices in the Arab world and; 2) Gain an overview of Barriers and Facilitators that may influence	(a) Palestine, Egypt, Sudan, the UAE, Qatar, Jordan, Lebanon, Saudi Arabia,	1

Author (first) year	Search: (a) Years (b) Region searched/ Targeted	Funding source indicated	Inclusion: (a) Technology/comparators (b) Type of studies included	Analysis: (a) Meta-analysis (b) Methods for quality assessment	Key Review topic	(a) Countries included (b) Applicability*	AMSTAR score[1]
					Arab women's BCS activities	Yemen, Israel, Turkey, Iran (b) Unclear	
Whelehan, 2013 [19]	(a) 2012 (b) world	the National Awareness and Early Diagnosis Initiative, under the auspices of the National Cancer Research Institute	(a) Pain during MM vs. no pain (b) observational and experimental (excluding RCT)	(a) Yes (b) Risk of bias, individual elements of quality	1. What is the range, nature and quality of the current evidence? 2. How commonly do women choose not to re-attend for BCS because of a prior painful MM? 3. Are there any sub-groups of women who are more likely to avoid BCS because of MM pain?	(a) The UK, NZ, the NL, the USA (b) HIC	4
Damiani, 2015 [20]	(a) 2000-2013 (b) HIC	None	(a) MM within a 2-year period among women with high and low level of education (b) Any design is searched; cross-sectional are included	(a) Yes (b) Risk of bias and quality by the criteria established by the researchers	Impact of women's educational attainment level on their screening adherence, by analysing the findings of the existing studies through a meta-analytic approach in order to increase the statistical power for this association and provide stronger evidence to policy makers for the decision making processes	(a) The USA, Mexico, Italy, South Korea, Spain (b) Upper-middle and HIC	4

Author (first) year	Search: (a) Years (b) Region searched/ Targeted	Funding source indicated	Inclusion: (a) Technology/comparators (b) Type of studies included	Analysis: (a) Meta-analysis (b) Methods for quality assessment	Key Review topic	(a) Countries included (b) Applicability*	AMSTAR score[1]
Diaz, 2017 [21]	(a) 1991 -2016<br>(b) world	Australia's National Health and Medical Research Council, the Strategic Research Partnership to Improve Cancer Control for Indigenous Australians, Cancer Council	(a) MM or Pap smear<br>(b) Original research	(a) Yes<br>(b) Risk of bias (Newcastle-Ottawa Scale)	To estimate the odds of participation in screening for breast cancer and cervical cancer, separately, for women with comorbidity compared to those without comorbidity	(a) The USA, Australia, Canada, Denmark, Spain<br>(b) HIC	5
Bhargava, 2018[28]	(a) 2016<br>(b) world (ethnic minorities)	Norwegian Breast Cancer Society	(a) MM<br>(b) Meta-analysis: studies with a control group	(a) Yes<br>(b) Not stated	Potential inequality in MM screening attendance among immigrant/ minorities and other women	(a) the USA, the UK, Australia, Canada, Denmark, Germany, the NL, NZ, Sweden, Switzerland	3
De Cuevas, 2018[29]	(a) 2018<br>(b) world /South Asian population	the National Institute for Health Research Collaboration for Leadership in Applied Health Research and Care North West Coast	(a) BCS (excluding BSE and CBE)<br>(b) Not specified	(a) No<br>(b) Critical Appraisal Skills Programme checklist	Cultural, social, structural and behavioural factors that influence asymptomatic breast and cervical cancer screening rates in South Asian populations	(a) Inductive: Canada (4), Spain; predictive: USA (9), Canada (4), Australia; Comparative: Singapore, USA, Malaysia, Spain, Canada	3

Author (first) year	Search: (a) Years (b) Region searched/ Targeted	Funding source indicated	Inclusion: (a) Technology/comparators (b) Type of studies included	Analysis: (a) Meta-analysis (b) Methods for quality assessment	Key Review topic	(a) Countries included (b) Applicability*	AMSTAR score[1]
Mathioudakis, 2019[30]	(a) 2018 (b) OECD/Europe	Cochrane Collaboration/ European Respiratory Society	(c) BCS (d) Qualitative and quantitative	(a) No (b) GRADE (qualitative and Critical Appraisal Skills Programme checklist (quantitative)	Values and preferences of women regarding decision making on breast cancer screening and diagnosis	Overdiagnosis: Europe (4), Australia; Not structured for the others	
**Reviews focused on benefits or benefits and harms of screening**							
Njor, 2012 [23]	(a) 2011 (b) Europe / Europe	National Monitoring Italian Centre	(a) MM vs no screening (b) Population-based studies	(a) Yes (b) No systematic methods. Some aspects of quality (lead time address and limitations of the studies such as use of registers for BC death are discussed)	Incidence-based mortality of service MM screening in Europe	(a) Denmark, Norway, Sweden, Italy, Spain, Finland (b) Western Europe	2
Abdel-Aleem, 2016 [24]	(a) 2014 (b) world	None	(a) Mobile clinics (b) Individual- and cluster-RCTs and non-RCTs	(a) Yes (b) Risk of bias, using the Cochrane EPOC criteria and the seven standard criteria for ITS studies	To evaluate the impact of mobile clinic services on women's and children's health	(a) The USA (b) The USA	11

Author (first) year	Search: (a) Years (b) Region searched/ Targeted	Funding source indicated	Inclusion: (a) Technology/comparators (b) Type of studies included	Analysis: (a) Meta-analysis (b) Methods for quality assessment	Key Review topic	(a) Countries included (b) Applicability*	AMSTAR score[1]
Hamashima, 2016 [25]	(a) 2012<br>(b) world, Japan / Japan	National Cancer Centre, Tokyo, Japan	(a) MM with and without CBE vs no screening, CBE alone and US with and without MM<br>(b) MM: RCT; CBE, US: observational, RCT	(a) Yes<br>(b) Methods are not described	Efficacy and/or effectiveness of MM with and without CBE, CBE alone and US with and without MM for BCS	(a) MM: Sweden, the UK; MM+CBE: the USA, Canada, Scotland; Not for the meta-analysis (on CBE, US)<br>(b) HIC (meta-analysis based on RCT), unclear (observational)	4
Chen, 2017 [26]	(a) 2015<br>(b) world/ Asia	Ministry of Science and Technology, Taiwan	(a) MM screening versus no screening<br>(b) RCT	(a) Yes<br>(b) Jadad	To clarify the debate on MM screening when used at the population level by elucidating a cascade of causal relationships between the 2 main parameters of participation rate and sensitivity, and the outcome of advanced BC and BC mortality based on the Bayesian meta-analysis and causal model	(a) The USA, Canada, the UK, Sweden<br>(b) HIC	4
Greenwald, 2017 [27]	(a) 2015<br>(b) world	None	(a) Mobile screening unit<br>(b) Any studies	(a) No	Evidence on the implementation of	(a) The USA, the UK, Sweden,	6

Author (first) year	Search: (a) Years (b) Region searched/ Targeted	Funding source indicated	Inclusion: (a) Technology/comparators (b) Type of studies included	Analysis: (a) Meta-analysis (b) Methods for quality assessment	Key Review topic	(a) Countries included (b) Applicability*	AMSTAR score[1]
				(b) Mixed Methods Appraisal Tool	MSUs for early cancer detection worldwide.	Australia, Italy, Canada, Belgium, France, Saudi Arabia, South Africa, Brazil, Taiwan, Mexico, Thailand, Greece (b) Unclear	

[1] AMSTAR – AMSTAR stands for A MeaSurement Tool to Assess systematic Reviews (https://amstar.ca). The AMSTAR checklist consists of 11 questions, with answers "ÿes" to each question accounted with a score of one. AMSTAR characterises quality at three levels: 8 to 11 is high quality, 4 to 7 is medium quality, 0 to 3 is low quality[33]; [2]The clusters unite articles that are related to each other by authorship or methodology.

BC – breast cancer; BSE – breast self-examination; CASP - Critical Appraisal Skills Programme; CBE – clinical breast examination; CBHWs - community-based health worker interventions; CONSORT - Consolidated Standards of Reporting Trials; GP - general practitioner; HIC – high-income countries; ITS - interrupted time series; MM – mammography; MSU - mobile screening unit; the NL – the Netherlands; NZ – the New Zealand; RCTs – randomized controlled trials; STROBE - The Strengthening the Reporting of Observational Studies in Epidemiology; UAE - the United Arab Emirates; US – ultrasonography; UK – United Kingdom; USA – United States of America.

References to the appendix C

1. Wagner TH. The effectiveness of mailed patient reminders on mammography screening: a meta-analysis. *Am J Prev Med* 1998;**14**: 64-70.
2. Sin JP, St Leger AS. Interventions to increase breast screening uptake: do they make any difference? *J Med Screen* 1999;**6**: 170-81.
3. J Jepson R, Clegg A, Forbes C, Lewis R, Sowden A, Kleijnen J. The determinants of screening uptake and interventions for increasing uptake: a systematic review. *Health Technol Assess* 2000;**4**: i-vii, 1-133.
4. Bonfill Cosp X, Marzo Castillejo M, Pladevall Vila M, Marti J, Emparanza José I. Strategies for increasing the participation of women in community breast cancer screening Cochrane Database of Systematic Reviews: John Wiley & Sons, Ltd, 2001.
5. Ratner PA, Bottorff JL, Johnson JL, Cook R, Lovato CY. A meta-analysis of mammography screening promotion. *Cancer Detect Prev* 2001;**25**: 147-60.
6. Denhaerynck K, Lesaffre E, Baele J, Cortebeeck K, Van Overstraete E, Buntinx F. Mammography screening attendance: meta-analysis of the effect of direct-contact invitation. *Am J Prev Med* 2003;**25**: 195-203.
7. O'Malley AS, Gonzalez RM, Sheppard VB, Huerta E, Mandelblatt J. Primary care cancer control interventions including Latinos. *American Journal of Preventive Medicine* 2003;**25**: 264-71.
8. Sohl SJ, Moyer A. Tailored interventions to promote mammography screening: a meta-analytic review. *Preventive medicine* 2007;**45**: 252-61.

9. Vernon SW, McQueen A, Tiro JA, del Junco DJ. Interventions to promote repeat breast cancer screening with mammography: a systematic review and meta-analysis. *Journal of the National Cancer Institute* 2010;**102**: 1023-39.

10. Brouwers MC, De Vito C, Bahirathan L, Carol A, Carroll JC, Cotterchio M, Dobbins M, Lent B, Levitt C, Lewis N, McGregor SE, Paszat L, et al. Effective interventions to facilitate the uptake of breast, cervical and colorectal cancer screening: an implementation guideline. *Implement Sci* 2011;**6**: 112.

11. Edwards AG, Naik G, Ahmed H, Elwyn GJ, Pickles T, Hood K, Playle R. Personalised risk communication for informed decision making about taking screening tests. *The Cochrane database of systematic reviews* 2013: Cd001865.

12. Bellhouse S, McWilliams L, Firth J, Yorke J, French DP. Are community-based health worker interventions an effective approach for early diagnosis of cancer? A systematic review and meta-analysis. *Psychooncology* 2017.

13. Curbow B, Bowie J, Garza MA, McDonnell K, Scott LB, Coyne CA, Chiappelli T. Community-based cancer screening programs in older populations: making progress but can we do better? *Preventive medicine* 2004;**38**: 676-93.

14. Soler-Michel P, Courtial I, Bremond A. [Reattendance of women for breast cancer screening programs. A review]. *Rev Epidemiol Sante Publique* 2005;**53**: 549-67.

15. Brewer NT, Salz T, Lillie SE. Systematic review: the long-term effects of false-positive mammograms. *Annals of internal medicine* 2007;**146**: 502-10.

16. Autier P, Hery C, Haukka J, Boniol M, Byrnes G. Advanced breast cancer and breast cancer mortality in randomized controlled trials on mammography screening. *J Clin Oncol* 2009;**27**: 5919-23.

17. Asadzadeh VF, Broeders MJ, Kiemeney LA, Verbeek AL. Opportunity for breast cancer screening in limited resource countries: a literature review and implications for Iran. *Asian Pacific journal of cancer prevention : APJCP* 2011;**12**: 2467-75.

18. Donnelly TT, Al Khater A-H, Al-Bader SB, Al Kuwari MG, Al-Meer N, Malik M, Singh R, Jong FC-D. Arab Women's Breast Cancer Screening Practices: A Literature Review. *Asian Pacific Journal of Cancer Prevention* 2013;**14**: 4519-28.

19. Whelehan P, Evans A, Wells M, Macgillivray S. The effect of mammography pain on repeat participation in breast cancer screening: a systematic review. *Breast* 2013;**22**: 389-94.

20. Damiani G, Basso D, Acampora A, Bianchi CB, Silvestrini G, Frisicale EM, Sassi F, Ricciardi W. The impact of level of education on adherence to breast and cervical cancer screening: Evidence from a systematic review and meta-analysis. *Preventive medicine* 2015;**81**: 281-9.

21. Diaz A, Kang J, Moore SP, Baade P, Langbecker D, Condon JR, Valery PC. Association between comorbidity and participation in breast and cervical cancer screening: A systematic review and meta-analysis. *Cancer Epidemiol* 2017;**47**: 7-19.

22. Kösters Jan P, Gøtzsche Peter C. Regular self-examination or clinical examination for early detection of breast cancer Cochrane Database of Systematic Reviews: John Wiley & Sons, Ltd, 2003.

23. Njor S, Nystrom L, Moss S, Paci E, Broeders M, Segnan N, Lynge E. Breast cancer mortality in mammographic screening in Europe: a review of incidence-based mortality studies. *Journal of medical screening* 2012;**19 Suppl 1**: 33-41.

24. Abdel-Aleem H, El-Gibaly OM, El-Gazzar AF, Al-Attar GS. Mobile clinics for women's and children's health. *Cochrane Database Syst Rev* 2016;**8**: Cd009677.

25. Hamashima C, Hamashima CC, Hattori M, Honjo S, Kasahara Y, Katayama T, Nakai M, Nakayama T, Morita T, Ohta K, Ohnuki K, Sagawa M, et al. The Japanese Guidelines for Breast Cancer Screening. *Japanese journal of clinical oncology* 2016;**46**: 482-92.

26. Chen TH, Yen AM, Fann JC, Gordon P, Chen SL, Chiu SY, Hsu CY, Chang KJ, Lee WC, Yeoh KG, Saito H, Promthet S, et al. Clarifying the debate on population-based screening for breast cancer with mammography: A systematic review of randomized controlled trials on mammography with Bayesian meta-analysis and causal model. *Medicine (Baltimore)* 2017;**96**: e5684.

27. Greenwald ZR, El-Zein M, Bouten S, Ensha H, Vazquez FL, Franco EL. Mobile Screening Units for the Early Detection of Cancer: A Systematic Review. *Cancer Epidemiol Biomarkers Prev* 2017;**26**: 1679-94.

28. Bhargava S, Moen K, Qureshi SA, Hofvind S. Mammographic screening attendance among immigrant and minority women: a systematic review and meta-analysis. Acta Radiol Stockh Swed 1987. 2018 Nov;59(11):1285–91.

29. Anderson de Cuevas RM RM, Saini P, Roberts D, Beaver K, Chandrashekar M, Jain A, et al. A systematic review of barriers and enablers to South Asian women's attendance for asymptomatic screening of breast and cervical cancers in emigrant countries. BMJ Open. 2018 Jul;8(7):e020892.

30. Mathioudakis AG, Salakari M, Pylkkanen L, Saz-Parkinson Z, Bramesfeld A, Deandrea S, et al. Systematic review on women's values and preferences concerning breast cancer screening and diagnostic services. Psychooncology. 2019 May;28(5):939–47.

31. Ferroni E, Camilloni L, Jimenez B, Furnari G, Borgia P, Guasticchi G, Giorgi Rossi P. How to increase uptake in oncologic screening: a systematic review of studies comparing population-based screening programs and spontaneous access. *Preventive medicine* 2012;**55**: 587-96.

32. Camilloni L, Ferroni E, Cendales BJ, Pezzarossi A, Furnari G, Borgia P, Guasticchi G, Giorgi Rossi P. Methods to increase participation in organised screening programs: a systematic review. *BMC Public Health* 2013;**13**: 464.

33. Shea BJ, Hamel C, Wells GA, Bouter LM, Kristjansson E, Grimshaw J, Henry DA, Boers M. AMSTAR is a reliable and valid measurement tool to assess the methodological quality of systematic reviews. *Journal of clinical epidemiology* 2009;**62**: 1013-20.

Appendix D. Quality and limitations of the systematic reviews and original studies

Author/ year	Original studies' limitations (as reported in the reviews)	Quality assessment by AMSTAR check-list											
		1. 'a priori' design [1]	2. Study selection [2]	3. Search [3]	4. grey literature [4]	5. Included / excluded [5]	6. Studies description [6]	7. Quality assessed [7]	8. Quality conclusions [8]	9. Combining results [9]	10. Publication bias [10]	11. Conflict of interest [11]	AMSTAR score [12]
Wagner, 1998 [1]	Only four studies were conducted outside of the USA. Methodological limitations of the studies are not mentioned.	N	C.t	Y	N	N	N	N	N	Y	N	N	2
Sin, 1999 [2]	The studies reliability, graded by quality of the design, varied (graded from I to IV)	N	N	Y	Y	N	Y	Y	N	NA	N	N	4
Jepson, 2000 [3]		N	N	Y	Y	N	Y	Y	Y	Y	N	N	6
Bonfill, 2001[4]	Methodologically weak studies were excluded from the review. Risk of bias for each of the included studies varies, no general summary on quality is provided.	Y	Y	Y	C.t	Y	Y	Y	C.t	Y	N	N	7
Ratner, 2001 [5]		N	C.t	N	N	N	Y	N	N	Y	N	N	2
Denhaerynck, 2003 [6]	Only four studies were conducted outside of the USA and had high heterogeneity; the proportions of women eligible for initial and repeat MM are rarely reported.	N	N	Y	Y	Y	Y	Y	C.t	Y	Y	N	7
O'Malley, 2003 [7]		N	C.t	Y	Y	N	Y	N	N	Y	N	N	4
Curbow, 2004 [9]		Y	N	Y	N	Y	Y	N	N	N	N	N	3

Author/ year	Original studies' limitations (as reported in the reviews)	Quality assessment by AMSTAR check-list											
		1. 'a priori' design [1]	2. Study selection [2]	3. Search [3]	4. grey literature [4]	5. Included / excluded [5]	6. Studies description [6]	7. Quality assessed [7]	8. Quality conclusions [8]	9. Combining results [9]	10. Publication bias [10]	11. Conflict of interest [11]	AMSTAR score [12]
Soler-Michel, 2005 [10]	Different limitations, table 1.	N	N	N	Y	N	Y	N	N	N	N	N	3
Brewer, 2007 [11]	Correlational study designs, a lack of clinical validation for multiple measurements, and possible heterogeneity.	N	Y	Y	Y	N	Y	C.t	N	Y	Y	N	5
Sohl, 2007 [12]	Small number of studies, only four of which delivered their interventions in-person	N	C.t	Y	N	N	Y	N	N	Y	Y	N	4
Autier, 2009 [13]	RCTs reported different markers of advanced disease. No quality assessment for the included studies was reported.	N	N	N	N	N	Y	N	N	C.t	N	N	1
Vernon, 2010 [14]	Quality of the included studies was variable. Among the main limitations: underreporting on the sample size calculation, not doing ITT analyses, not adjusting for the effect of nested samples, not comparing characteristics of the respondents and non-respondents at the baseline, or not comparing the final sample with dropouts, not testing for differential attrition. Not all studies compared participants with dropouts or reported differential attrition by study group, which may result in overestimated effect sizes.	N	Y	Y	N	Y	Y	Y	Y	Y	Y	N	8
Asadzadeh, 2011 [15]		N	C.t	Y	N	N	N	N	N	N	N	N	1

Author/ year	Original studies' limitations (as reported in the reviews)	Quality assessment by AMSTAR check-list											
		1. 'a priori' design [1]	2. Study selection [2]	3. Search [3]	4. grey literature [4]	5. Included / excluded [5]	6. Studies description [6]	7. Quality assessed [7]	8. Quality conclusions [8]	9. Combining results [9]	10. Publication bias [10]	11. Conflict of interest [11]	AMSTAR score [12]
Brouwers, 2011 [16]	The quality of evidence ranges from weak to excellent. Poor reporting and so questionable quality of the included RCTs with possibility of bias.	Y	C.t	Y	N	N	Y	Y	Y	N	N	N	5
Njor, 2012 [17]	Healthy user bias and lead time biases in some studies (by which MR can be underestimated); biased comparison of groups in some studies; referral to intent-to-treat analysis may be missing.	N	Y	N	N	N	Y	N	N	N	N	N	2
Donnelly, 2013 [18]		N	N	Y	N	N	N	N	N	N	N	N	1
Edwards, 2013 [19]	Only nine studies met four or more of the nine criteria for low risk of bias.	Y	Y	Y	N	Y	Y	Y	Y	Y	N	Y	9
Whelehan, 2013 [20]		N	Y	Y	N	N	Y	N	N	Y	N	N	4
Damiani, 2015 [21]	The methodological quality was assessed as "moderate" for all included studies.	N	Y	N	N	N	Y	Y	N	Y	N	N	4
Abdel-Aleem, 2016 [22]		N	Y	Y	Y	Y	Y	Y	Y	Y	Y	N	11
Hamashima, 2016 [23]	Methodologic limitations in all eight RCT for MM (all included into meta-analysis); quality is	N	Y	Y	N	N	Y	N	N	Y	N	N	4

Author/ year	Original studies' limitations (as reported in the reviews)	Quality assessment by AMSTAR check-list											
		1. 'a priori' design [1]	2. Study selection [2]	3. Search [3]	4. grey literature [4]	5. Included / excluded [5]	6. Studies description [6]	7. Quality assessed [7]	8. Quality conclusions [8]	9. Combining results [9]	10. Publication bias [10]	11. Conflict of interest [11]	AMSTAR score [12]
	not discussed and not mentioned for the studies on CBE and US.												
Bellhouse, 2017 [24]	Methodological quality of the studies was poor; the risk of bias was depicted as well.	N	N	Y	N	N	Y	N	N	Y	Y	N	4
Chen, 2017 [25]	Lack of information on contamination of the control group.	Y	C.t.	Y	N	N	Y	C.t	C.t	Y	N	N	4
Diaz, 2017 [26]	The few included studies that were assessed to have low risk of bias. Studies generally were unable to distinguish screening MM from those undertaken for diagnostic investigation or post-diagnosis surveillance, and most studies did not attempt to minimise the risk of misclassification.	N	C.t.	Y	N	N	Y	Y	Y	Y	N	N	5
Greenwald, 2017 [27]	Most biases were related to incomplete outcomes (all studies design); the other biases: appropriate measures and selection bias (quantitative design) and selection bias and confounding (observational design).	Y	C.t.	Y	Y	Y	Y	N	N	Y	N	N	6
Bhargava, 2018 [28]		N	C.t.	Y	N	N	Y	N	N	Y	N	N	3

**Author/ year**	**Original studies' limitations (as reported in the reviews)**	**Quality assessment by AMSTAR check-list**											
		**1. 'a priori' design [1]**	**2. Study selection [2]**	**3. Search [3]**	**4. grey literature [4]**	**5. Included / excluded [5]**	**6. Studies description [6]**	**7. Quality assessed [7]**	**8. Quality conclusions [8]**	**9. Combining results [9]**	**10. Publication bias [10]**	**11. Conflict of interest [11]**	**AMSTAR score [12]**
De Cuevas, 2018 [29]	Poverty of theory and methodological limitations (poor sampling techniques, variability in definitions of a South Asian population, lack of objectivity in recording)	N	N	Y	Y	N	Y	C.t.	C.t.	N	N	N	3
Mathioudakis, 2019[30]	No confidence that the participants of several of the included studies received balanced information in order to understand complex concepts, such as overdiagnosis	Y	N	N	N	N	Y	N	N	N	N	N	2
Cluster articles Camilloni and Ferroni (2012-2013)													
Ferroni, 2012 [31]	Methodologically week studies were excluded from the review. In the included studies, the definition of usual care did not appear to be the same for all authors.	Y	N	Y	Y	N	N	C.t	C.t	Y	N	N	4
Camilloni, 2013 [32]	Poor reporting of the studies, including randomisation and allocation, follow-up, and funding. Various methodologic limitations, including ethics (lack of consent for participation). Scarcity of sound studies measuring the effect of mass media campaigns and community-based interventions. For all studies on community education interventions, analyses did not adequately take into account cluster randomization.	Y	N	Y	Y	Y	Y	Y	Y	Y	N	N	8

*The clusters unite articles are those related to each other by authorship or methodology.

AMSTAR – AMSTAR stands for A MeaSurement Tool to Assess systematic Reviews (https://amstar.ca). The AMSTAR checklist consists of 11 questions, with answers "ÿes" to each question accounted with a score of one. [1] 'a priori' design; [2] duplicate study selection; [3] comprehensive search; [4] grey literature searched; [5] A list of studies included and excluded; [6] Characteristics of the included studies; [7] Scientific quality of the studies assessed; [8] Scientific quality of the studies is included in conclusions; [9] Methods to combine the results are appropriate; [10] Likelihood of publication bias; [11] Conflict of interest for the review and the included studies; [12] AMSTAR total score. AMSTAR characterises quality at three levels: 8 to 11 is high quality, 4 to 7 is medium quality, 0 to 3 is low quality.

Abbreviations: CBE – clinical breast examination; C.t – cannot tell; ITT - intention-to-treat; MM – mammography; MR – mortality reduction; RCT – randomized controlled trial; US – ultrasound; the USA – the United States of America.

References to the appendix D

1. Wagner TH. The effectiveness of mailed patient reminders on mammography screening: a meta-analysis. *Am J Prev Med* 1998;**14**: 64-70.
2. Sin JP, St Leger AS. Interventions to increase breast screening uptake: do they make any difference? *J Med Screen* 1999;**6**: 170-81.
3. Jepson R, Clegg A, Forbes C, Lewis R, Sowden A, Kleijnen J. The determinants of screening uptake and interventions for increasing uptake: a systematic review. Health Technol Assess 2000;4: i-vii, 1-133.
4. Bonfill Cosp X, Marzo Castillejo M, Pladevall Vila M, Marti J, Emparanza José I. Strategies for increasing the participation of women in community breast cancer screening Cochrane Database of Systematic Reviews: John Wiley & Sons, Ltd, 2001.
5. Ratner PA, Bottorff JL, Johnson JL, Cook R, Lovato CY. A meta-analysis of mammography screening promotion. *Cancer Detect Prev* 2001;**25**: 147-60.
6. Denhaerynck K, Lesaffre E, Baele J, Cortebeeck K, Van Overstraete E, Buntinx F. Mammography screening attendance: meta-analysis of the effect of direct-contact invitation. *Am J Prev Med* 2003;**25**: 195-203.
7. O'Malley AS, Gonzalez RM, Sheppard VB, Huerta E, Mandelblatt J. Primary care cancer control interventions including Latinos: a review. *Am J Prev Med* 2003;**25**: 264-71.
8. Kösters JP, Gøtzsche PC. Regular self-examination or clinical examination for early detection of breast cancer Cochrane Database of Systematic Reviews: John Wiley & Sons, Ltd, 2003.
9. Curbow B, Bowie J, Garza MA, McDonnell K, Scott LB, Coyne CA, Chiappelli T. Community-based cancer screening programs in older populations: making progress but can we do better? *Preventive medicine* 2004;**38**: 676-93.
10. Soler-Michel P, Courtial I, Bremond A. [Reattendance of women for breast cancer screening programs. A review]. *Rev Epidemiol Sante Publique* 2005;**53**: 549-67.
11. Brewer NT, Salz T, Lillie SE. Systematic review: the long-term effects of false-positive mammograms. *Annals of internal medicine* 2007;**146**: 502-10.
12. Sohl SJ, Moyer A. Tailored interventions to promote mammography screening: a meta-analytic review. *Preventive medicine* 2007;**45**: 252-61.
13. Autier P, Hery C, Haukka J, Boniol M, Byrnes G. Advanced breast cancer and breast cancer mortality in randomized controlled trials on mammography screening. *J Clin Oncol* 2009;**27**: 5919-23.
14. Vernon SW, McQueen A, Tiro JA, del Junco DJ. Interventions to promote repeat breast cancer screening with mammography: a systematic review and meta-analysis. *Journal of the National Cancer Institute* 2010;**102**: 1023-39.
15. Asadzadeh VF, Broeders MJ, Kiemeney LA, Verbeek AL. Opportunity for breast cancer screening in limited resource countries: a literature review and implications for Iran. *Asian Pac J Cancer Prev* 2011;**12**: 2467-75.
16. Brouwers MC, De Vito C, Bahirathan L, Carol A, Carroll JC, Cotterchio M, Dobbins M, Lent B, Levitt C, Lewis N, McGregor SE, Paszat L, et al. What implementation interventions increase cancer screening rates? a systematic review. *Implement Sci* 2011;**6**: 111.
17. Njor S, Nystrom L, Moss S, Paci E, Broeders M, Segnan N, Lynge E. Breast cancer mortality in mammographic screening in Europe: a review of incidence-based mortality studies. *J Med Screen* 2012;**19 Suppl 1**: 33-41.
18. Donnelly TT, Khater AH, Al-Bader SB, Al Kuwari MG, Al-Meer N, Malik M, Singh R, Jong FC. Arab women's breast cancer screening practices: a literature review. *Asian Pac J Cancer Prev* 2013;**14**: 4519-28.

19. Edwards AG, Naik G, Ahmed H, Elwyn GJ, Pickles T, Hood K, Playle R. Personalised risk communication for informed decision making about taking screening tests. *The Cochrane database of systematic reviews* 2013: Cd001865.

20. Whelehan P, Evans A, Wells M, Macgillivray S. The effect of mammography pain on repeat participation in breast cancer screening: a systematic review. *Breast* 2013;**22**: 389-94.

21. Damiani G, Basso D, Acampora A, Bianchi CB, Silvestrini G, Frisicale EM, Sassi F, Ricciardi W. The impact of level of education on adherence to breast and cervical cancer screening: Evidence from a systematic review and meta-analysis. *Preventive medicine* 2015;**81**: 281-9.

22. Abdel-Aleem H, El-Gibaly OM, El-Gazzar AF, Al-Attar GS. Mobile clinics for women's and children's health. *Cochrane Database Syst Rev* 2016;**8**: Cd009677.

23. Hamashima C, Hamashima CC, Hattori M, Honjo S, Kasahara Y, Katayama T, Nakai M, Nakayama T, Morita T, Ohta K, Ohnuki K, Sagawa M, et al. The Japanese Guidelines for Breast Cancer Screening. *Japanese journal of clinical oncology* 2016;**46**: 482-92.

24. Bellhouse S, McWilliams L, Firth J, Yorke J, French DP. Are community-based health worker interventions an effective approach for early diagnosis of cancer? A systematic review and meta-analysis. *Psychooncology* 2017.

25. Chen TH, Yen AM, Fann JC, Gordon P, Chen SL, Chiu SY, Hsu CY, Chang KJ, Lee WC, Yeoh KG, Saito H, Promthet S, et al. Clarifying the debate on population-based screening for breast cancer with mammography: A systematic review of randomized controlled trials on mammography with Bayesian meta-analysis and causal model. *Medicine (Baltimore)* 2017;**96**: e5684.

26. Diaz A, Kang J, Moore SP, Baade P, Langbecker D, Condon JR, Valery PC. Association between comorbidity and participation in breast and cervical cancer screening: A systematic review and meta-analysis. *Cancer Epidemiol* 2017;**47**: 7-19.

27. Greenwald ZR, El-Zein M, Bouten S, Ensha H, Vazquez FL, Franco EL. Mobile Screening Units for the Early Detection of Cancer: A Systematic Review. *Cancer Epidemiol Biomarkers Prev* 2017;**26**: 1679-94.

28. Bhargava S, Moen K, Qureshi SA, Hofvind S. Mammographic screening attendance among immigrant and minority women: a systematic review and meta-analysis. Acta Radiol Stockh Swed 1987. 2018 Nov;59(11):1285–91.

29. Anderson de Cuevas RM RM, Saini P, Roberts D, Beaver K, Chandrashekar M, Jain A, et al. A systematic review of barriers and enablers to South Asian women’s attendance for asymptomatic screening of breast and cervical cancers in emigrant countries. BMJ Open. 2018 Jul;8(7):e020892.

30. Mathioudakis AG, Salakari M, Pylkkanen L, Saz-Parkinson Z, Bramesfeld A, Deandrea S, et al. Systematic review on women’s values and preferences concerning breast cancer screening and diagnostic services. Psychooncology. 2019 May;28(5):939–47.

31. Ferroni E, Camilloni L, Jimenez B, Furnari G, Borgia P, Guasticchi G, Giorgi Rossi P. How to increase uptake in oncologic screening: a systematic review of studies comparing population-based screening programs and spontaneous access. *Preventive medicine* 2012;**55**: 587-96.

32. Camilloni L, Ferroni E, Cendales BJ, Pezzarossi A, Furnari G, Borgia P, Guasticchi G, Giorgi Rossi P. Methods to increase participation in organised screening programs: a systematic review. *BMC Public Health* 2013;**13**: 464.

1 **Appendix E. Outcomes and definitions reported in systematic reviews**

Author/year	Outcome range	Outcome definition	Factors impacting the outcome
Wagner, 1998 [1]	MM usage: average 41% outside of the USA, 43% in the USA	The percentage of women who received MM	Positive effect: mailed patient reminders.<br>Possible (1 study): reminders with fixed appointment, time since visit, income, education, being married, time off from work, health status, family history, and previous mammography<br>Not clear (inconsistent): tailored letters vs. generic reminders, age, race, insurance, travel distance.<br>No effect: type of insurance, English as a second language, number of visits in the previous year.
Chen, 2017 [25]	Attendance rate: 61.3% - 90.4%.	Number of attendees divided by the number of invited subjects.	Age and socioeconomic status, method of invitation (Canadian trial) (Mentions but does not explore)
Bonfill, 2001 [4]	PR (no definition)	Attendance achieved in the groups exposed to recruitment strategies	Positive effect: most active recruitment strategies for BCS programs (letter of invitation, mailed educational material, letter of invitation plus phone call, phone call, and training activities plus direct reminders for the women).<br>No effect: home visits.<br>Negative/not clear effect: letters of invitation to multiple examinations plus educational material.
Brouwers, 2011 [16]	Uptake: 18-61%	The screening rate	Positive effect: client reminders, small media, one-on-one education, provider audit and feedback, reducing structural barriers<br>Insufficient evidence: client reminders, client incentives, mass media alone, group education, reducing out-of-pocket costs, provider incentives.
Hamashima, 2016 [23]	PR: 74-85%	No definition	No information
Bellhouse, 2017 [24]	Adherence/uptake (not clear outcome)[4]: 16-92% for MM, 31-72% CBE, 27-45% BSE	The total numbers of participants who had been screened versus control condition	Positive effect: CBHW interventions (MM, BSE, CBE)
Edwards, 2013 [19]	Uptake (screening uptake, not isolated for BC): 53-59%	No definition	No impact of personalised risk communication versus general risk information for MM screening interventions OR 0.84 (95%CI 0.68, 1.03) (while among all screenings OR indicated weak evidence, consistent with a small effect)
Autier, 2009 [13]	Attendance (1st round): 67-87%	Women who attended at least one MM round	No information
Abdel-Aleem, 2016 [22]	Uptake: 40-55%	Utilisation, coverage, or access (used coverage and service utilisation as proxies)	Positive effect: access to mobile MM screening combined with health education.
O'Malley, 2003 [8]	PR: 11-87%	No definition. Definitions of the outcomes reported in the table 2 vary (mainly "receipt of the MM within 1-12 month after the intervention")	Positive effect: involve "in-reach" in community health clinics, patient-targeted (providing transportation, facilitated appointment scheduling, reduced costs or provided vouchers for screening tests (vs only bilingual instructions), provided one-on-one counselling, sent tailored letters, or gave reminders), provider-directed (office system provider prompts and reminders (the most effective), nurse-based interventions, clinic

Author/year	Outcome range	Outcome definition	Factors impacting the outcome
			reorganization, physician education, and audit with feedback. No impact (not reported systematically): academic detailing about cancer screening and prevention
Bhargava, 2018[28]	General attendance (immigrant vs non-immigrants) : 46.2% vs 55% (OR = 0.64, CI 0.56-0.73) ; Europe: 64.1% vs 77.6%, US: 48.9% vs 78.3%, Oceania : 42.8% vs 53%.	Screening attendance (whether women had MM)	Negative impact: immigrant vs non-immigrant.
Vernon, 2010 [14]	Repeat screening (calculated) weighted average: 63-72%, annual 62-72%, biennial 73-77%.	At least two consecutive, on-schedule MM during a given period; a certain number of MM during a given period; or at least two MM on an age-appropriate schedule.	Positive effect: education/motivation/ counselling (homogeneous in the effect size), reminders (OR 1.79, 95% CI(1.41 - 2.29), heterogeneous effect), reminder only vs education/motivation and counselling (heterogeneous) Unclear/no effect: study designs, methods, settings, populations (age <50), intervention strategies, delivery modes, outcome measurements, screening intervals, or use of theory.
Denhaerynck, 2003 [6]	Attendance: 6-78% (median, 39%)	The number screened divided to the size of the study groups (after the subtraction of follow-up loss)	Positive effect: direct-contact strategies (telephone calls, in-home educational interventions or visits by health workers). Possible impact of organizational features of a healthcare system. Higher gain: in the jurisdictions with the low coverage rate, in the group of non-responders as a result of an initial invitation vs primary population, those who were contacted by telephone vs those who were contacted in person. Lower gain: the group of studies targeting a population that generally underutilizes MM vs a more general population.
Whelehan, 2013 [20]	Repeat participation/re-attendance: not reporting pain – 72-74%, reporting pain – 65-73%	No definition	Negative effect: pain at MM (but not statistically significant, RR 1.34 (95% CI: 0.94-1.91)).
Curbow, 2004 [9]	Utilization MM: 39-68% (USA) , 7-13% (Singapore); BSE: 19-33% (USA), CBE: 68-74% (USA)	The percentage of women who reported a MM before and after an intervention	Positive effect: second mailed reminder vs call, educational session (BSE, MM), home visit (combined with reminder letter and mailed educational materials [Singapore]), No impact: media intervention, Not clear/contradictory: written materials and vouchers Utilization was higher among white than black low-income women
Sohl, 2007 [12]	Adherence: not reported absolute values	(a) Repeat/regular MM (when women reported a MM within 24 months of the survey and a prior MM within 24	Positive effect: tailored interventions, tailored interventions that used the Health Belief Model (but not Transtheoretical Model), interventions that included a physician recommendation. No /not significant effect: type of population recruited, participants' pre-intervention level of mammography

Author/year	Outcome range	Outcome definition	Factors impacting the outcome
		months of the most recent MM)<br>(b) Recent MM (when woman had had a MM within 24 months of the survey but had not had a prior MM within 24 months of the most recent."	adherence, no difference among interventions delivered in person, by telephone, or in print (similarly effective)<br>Negative effect: Tailoring an intervention according to the recipients' ethnicity
Sin, 1999 [2]	Uptake: 46-86% and 4-42% for non-attenders	The proportion of women invited for MM who actually attend	Positive effect: combination of invitations with fixed appointments (reminder letter, or telephone, or invitation letter with fixed appointment); reminder letter for non-attendants, reminder letters with fixed appointments.<br>Limited effect: phone reminders<br>No effect: endorsement of the invitation by a GP, extensive health education by the GP vs verbal recommendation, home visits vs. simple invitation approach, social network<br>Not enough evidence: bus transport to the screening centre, multistrategy interventions.
Jepson, 2000 [3]	Uptake: 16-88% (based on study, invitation technique)	The proportion of persons eligible to be screened within a population who have been both invited for screening and have received an adequate screen during a specified period	<u>Interventions aimed at individuals</u><br>Positive effect: invitation appointments, letters (less effective for MM) and telephone calls; telephone counselling; removal of financial barriers.<br>Possible positive effect: educational home visits; opportunistic screening; multicomponent community interventions; simpler procedures; combination of different components aimed at individuals; reminders for non-attenders; invitation follow-ups.<br>Limited effect: provided educational materials or sessions; risk-factor questionnaires; face-to-face counselling.<br>No effect: the use of rewards or incentives.<br>There was insufficient evidence to evaluate the effectiveness of other interventions.<br><u>Interventions aimed at healthcare workers</u><br>Positive effect: physician reminders.<br>Possible positive effect: office systems, audit and feedback.<br>Not enough evidence: physician education.<br><u>Interventions aimed at both individuals and physicians:</u><br>Positive effect: physician reminders combined with invitations to individuals.<br><u>Interventions aimed at individuals versus interventions aimed at physicians:</u> small beneficial effect.<br><u>Individual factors</u><br>Positive effect: expressed an intention to attend, previous MM, medical insurance.<br>Unclear direction of the effect: recommendation from a healthcare provider.<br>No effect: age, ethnicity, education, marital status, attendance for other screening tests, screening knowledge, medical history, perceived vulnerability and worries about screening.
Damiani, 2015 [21]	Adherence (Not clear outcome)[4]: 15-82%	Having at least one MM within a two year period	Positive effect: high level of education (heterogeneous), homogenous among women ≥50 y.o.

Author/year	Outcome range	Outcome definition	Factors impacting the outcome
Ratner, 2001 [5]	Screening rate/participation in MM/ screening uptake (calculated from the table): 9-48%	Not defined (assessed from the table 4): number of screened divided to the sample size of the study group	Positive effect: more recent studies, receiving the invitation<br>Negative effect: studies on older women and set in clinics<br>No impact: teaching on screening importance, enabling to attend, encouraging recommendations from physicians, reinforcement through rewards and incentives.
Greenwald, 2017 [27]	Screening rate: 44.3- 63.4% [5]: proportion of first-screen clients: 11-72%	No definition	Possible positive impact (not systematic): MSU; invitation by mail or telephone, publicity (flyers, radio, car loudspeaker, newspaper), word of mouth or physician referral, home visits.<br>Negative effect (not systematic): lack of awareness / information, negative beliefs or attitudes towards screening, fear of discomfort/embarrassment, cost.
Mathioudakis, 2019[30]	Not reported	Not reported	Not structured/synthesised:<br>Among elderly (80 or over) women (only 2 studies): perceived individual risk of BC, physician's advice, previous screening habits and experiences with MM, social and family influences, age, doctor's counselling.<br>Other: previous node biopsy
Diaz, 2017 [26]	Participation (within 1 year): 34-89%, other timeframe (including ever): 49-98%	Screening participation within a timeframe (denominator is not defined)	Negative effect: comorbidity
Soler-Michel, 2005 [10]	Re-attendance (secondary participation): 62-91%	Successive participations of women after their first screening	Positive effect: main: intention to participate, positive views about initial screening (punctuality and clinic staff courteous), psychological factors (being not afraid, perception of benefits, vulnerability etc.); others: practice of other preventive health behaviours, outside support from physicians, knowledge of BC and BCS, no payment required, younger age, being married and working, good knowledge of cancer and screening.<br>Negative effect: previous screening experience (pain, anxiety, embarrassment and screening organization).<br>No effect: other chronic diseases.<br>Not clear effect: having acquaintances with cancer, socio-economic status, level of education, access to screening and appointment time, perception of severity of cancer, self-believes.
Njor, 2012 [17]	PR: 71-88%	No definition	No information
Brewer, 2007 [11]	Re-attendance (calculated weighted average 76%): Europe - 71-94%, USA – 57-81%, Canada – 49-74%, after FP (calculated weighted average 64%): Europe 73-95%, USA – 62-87%, Canada – 27-52%	The proportion of women returning for routine MM who received FP results and those who received normal results	Negative/not clear effect: false-positive result of the previous MM.<br>Region of implementation had an impact on the direction of the effect of the previous false-positive MM (the USA - positive, Canada – negative, Europe -neutral).
Asadzadeh, 2011 [15]	Practice/ compliance/ attendance rate:	No definition	Negative effect: low level of education, occupational and socioeconomic status, young age of the target population, low awareness of breast cancer, social and cultural factors;

Author/year	Outcome range	Outcome definition	Factors impacting the outcome
	most <50% (range 10-80%)[3]		Asian women: lack of knowledge, socio-demographic factors and psychosocial factors.<br>No effect/not clear: knowledge and attitudes toward BCS.
Donnelly, 2013 [18]	Practice: BSE 4-27%, CBE 13-70%, MM 1-67% [3]	No definition (includes regular, not-specified, and ever timeline)	Positive effect: higher education, income, being married, employment, living in urban area, recommendation from a family/friend, positive attitude, self-care as a priority.<br>Not clear /dual side of the effect: religion, age, perceived effectiveness of BCS.<br>Negative effect: self-care being a low priority, fear of the diagnosis BC, pain from MM/CBE, perception of low susceptibility, perceived effectiveness of screening., cancer fear, embarrassment, low knowledge, costs, perception of low susceptibility to BC.
De Cuevas, 2018[29]	MM within 2 years: 18-64%, MM ever: 57-81%, CBE ever: 39%.	By studies (ever had BCS or had BCS within 2 years)	Inductive studies, important factors (narrative): knowledge/attitudes (consider it incurable, “white woman’s disease”), culture (stigma, family), cultural adaptation (needs for chaperone, being used to paid services).<br>Predictive studies (narrative, lower screening rates): no health insurance, younger women, lower level of education, lower knowledge in BC, self-reported barriers, less time spent in the host country.<br>Comparative studies: no long-term change in screening uptake but increase in knowledge
Camilloni, 2013[32]	Participation (range, calculated)[;6] 21-66%	Participated in in the first level test of the program versus total invited to be screened	Positive effect: letter or phone reminders (vs invitation letter only), GP signing the invitation (modest), scheduled appointment (vs. open appointment), reduction of logistical barriers (MSU), individual educational interventions<br>Negative effect: long letters (for people with low education)<br>Not clear effect: public information campaigns, face-to-face reminder, the addition of a gift to the standard invitation letter, letter for physician referral vs a letter for direct access, tailored letters vs personal letter, printed materials
Ferroni, 2012[31]	(a)Coverage /(b)participation (uptake, adherence, - no definition) (not clear):41-60%	No clear connection between the definitions and reported values (a) proportion of the target population who had had a test in the preceding two years or once in their life; (b) proportion of invited/ contacted people who actually underwent the test in the screening programme	Positive effect: the invitation letter or phone call, the invitation letter plus phone call, the invitation letter plus GP reminder, and GP-based interventions (GP reminders)<br>No effect: invitation letter-based versus GP-based organization

References to the appendix E

1. Wagner TH. The effectiveness of mailed patient reminders on mammography screening: a meta-analysis. *Am J Prev Med* 1998;**14**: 64-70.

2. Sin JP, St Leger AS. Interventions to increase breast screening uptake: do they make any difference? *J Med Screen* 1999;**6**: 170-81.

3. Jepson R, Clegg A, Forbes C, Lewis R, Sowden A, Kleijnen J. The determinants of screening uptake and interventions for increasing uptake: a systematic review. Health Technol Assess 2000;4: i-vii, 1-133.

4. Bonfill Cosp X, Marzo Castillejo M, Pladevall Vila M, Marti J, Emparanza José I. Strategies for increasing the participation of women in community breast cancer screening Cochrane Database of Systematic Reviews: John Wiley & Sons, Ltd, 2001.

5. Ratner PA, Bottorff JL, Johnson JL, Cook R, Lovato CY. A meta-analysis of mammography screening promotion. *Cancer Detect Prev* 2001;**25**: 147-60.

6. Denhaerynck K, Lesaffre E, Baele J, Cortebeeck K, Van Overstraete E, Buntinx F. Mammography screening attendance: meta-analysis of the effect of direct-contact invitation. *Am J Prev Med* 2003;**25**: 195-203.

7. O'Malley AS, Gonzalez RM, Sheppard VB, Huerta E, Mandelblatt J. Primary care cancer control interventions including Latinos: a review. *Am J Prev Med* 2003;**25**: 264-71.

8. Kösters JP, Gøtzsche PC. Regular self-examination or clinical examination for early detection of breast cancer Cochrane Database of Systematic Reviews: John Wiley & Sons, Ltd, 2003.

9. Curbow B, Bowie J, Garza MA, McDonnell K, Scott LB, Coyne CA, Chiappelli T. Community-based cancer screening programs in older populations: making progress but can we do better? *Preventive medicine* 2004;**38**: 676-93.

10. Soler-Michel P, Courtial I, Bremond A. [Reattendance of women for breast cancer screening programs. A review]. *Rev Epidemiol Sante Publique* 2005;**53**: 549-67.

11. Brewer NT, Salz T, Lillie SE. Systematic review: the long-term effects of false-positive mammograms. *Annals of internal medicine* 2007;**146**: 502-10.

12. Sohl SJ, Moyer A. Tailored interventions to promote mammography screening: a meta-analytic review. *Preventive medicine* 2007;**45**: 252-61.

13. Autier P, Hery C, Haukka J, Boniol M, Byrnes G. Advanced breast cancer and breast cancer mortality in randomized controlled trials on mammography screening. *J Clin Oncol* 2009;**27**: 5919-23.

14. Vernon SW, McQueen A, Tiro JA, del Junco DJ. Interventions to promote repeat breast cancer screening with mammography: a systematic review and meta-analysis. *Journal of the National Cancer Institute* 2010;**102**: 1023-39.

15. Asadzadeh VF, Broeders MJ, Kiemeney LA, Verbeek AL. Opportunity for breast cancer screening in limited resource countries: a literature review and implications for Iran. *Asian Pac J Cancer Prev* 2011;**12**: 2467-75.

16. Brouwers MC, De Vito C, Bahirathan L, Carol A, Carroll JC, Cotterchio M, Dobbins M, Lent B, Levitt C, Lewis N, McGregor SE, Paszat L, et al. What implementation interventions increase cancer screening rates? a systematic review. *Implement Sci* 2011;**6**: 111.

17. Njor S, Nystrom L, Moss S, Paci E, Broeders M, Segnan N, Lynge E. Breast cancer mortality in mammographic screening in Europe: a review of incidence-based mortality studies. *J Med Screen* 2012;**19 Suppl 1**: 33-41.

18. Donnelly TT, Khater AH, Al-Bader SB, Al Kuwari MG, Al-Meer N, Malik M, Singh R, Jong FC. Arab women's breast cancer screening practices: a literature review. *Asian Pac J Cancer Prev* 2013;**14**: 4519-28.

19. Edwards AG, Naik G, Ahmed H, Elwyn GJ, Pickles T, Hood K, Playle R. Personalised risk communication for informed decision making about taking screening tests. *The Cochrane database of systematic reviews* 2013: Cd001865.

20. Whelehan P, Evans A, Wells M, Macgillivray S. The effect of mammography pain on repeat participation in breast cancer screening: a systematic review. *Breast* 2013;**22**: 389-94.

21. Damiani G, Basso D, Acampora A, Bianchi CB, Silvestrini G, Frisicale EM, Sassi F, Ricciardi W. The impact of level of education on adherence to breast and cervical cancer screening: Evidence from a systematic review and meta-analysis. *Preventive medicine* 2015;**81**: 281-9.

22. Abdel-Aleem H, El-Gibaly OM, El-Gazzar AF, Al-Attar GS. Mobile clinics for women's and children's health. *Cochrane Database Syst Rev* 2016;**8**: Cd009677.

23. Hamashima C, Hamashima CC, Hattori M, Honjo S, Kasahara Y, Katayama T, Nakai M, Nakayama T, Morita T, Ohta K, Ohnuki K, Sagawa M, et al. The Japanese Guidelines for Breast Cancer Screening. *Japanese journal of clinical oncology* 2016;**46**: 482-92.

24. Bellhouse S, McWilliams L, Firth J, Yorke J, French DP. Are community-based health worker interventions an effective approach for early diagnosis of cancer? A systematic review and meta-analysis. *Psychooncology* 2017.

25. Chen TH, Yen AM, Fann JC, Gordon P, Chen SL, Chiu SY, Hsu CY, Chang KJ, Lee WC, Yeoh KG, Saito H, Promthet S, et al. Clarifying the debate on population-based screening for breast cancer with mammography: A systematic review of randomized controlled trials on mammography with Bayesian meta-analysis and causal model. *Medicine (Baltimore)* 2017;**96**: e5684.

26. Diaz A, Kang J, Moore SP, Baade P, Langbecker D, Condon JR, Valery PC. Association between comorbidity and participation in breast and cervical cancer screening: A systematic review and meta-analysis. *Cancer Epidemiol* 2017;**47**: 7-19.

27. Greenwald ZR, El-Zein M, Bouten S, Ensha H, Vazquez FL, Franco EL. Mobile Screening Units for the Early Detection of Cancer: A Systematic Review. *Cancer Epidemiol Biomarkers Prev* 2017;**26**: 1679-94.

28. Bhargava S, Moen K, Qureshi SA, Hofvind S. Mammographic screening attendance among immigrant and minority women: a systematic review and meta-analysis. Acta Radiol Stockh Swed 1987. 2018 Nov;59(11):1285–91.

29. Anderson de Cuevas RM RM, Saini P, Roberts D, Beaver K, Chandrashekar M, Jain A, et al. A systematic review of barriers and enablers to South Asian women's attendance for asymptomatic screening of breast and cervical cancers in emigrant countries. BMJ Open. 2018 Jul;8(7):e020892.

30. Mathioudakis AG, Salakari M, Pylkkanen L, Saz-Parkinson Z, Bramesfeld A, Deandrea S, et al. Systematic review on women's values and preferences concerning breast cancer screening and diagnostic services. Psychooncology. 2019 May;28(5):939–47.

31. Ferroni E, Camilloni L, Jimenez B, Furnari G, Borgia P, Guasticchi G, Giorgi Rossi P. How to increase uptake in oncologic screening: a systematic review of studies comparing population-based screening programs and spontaneous access. *Preventive medicine* 2012;**55**: 587-96.

32. Camilloni L, Ferroni E, Cendales BJ, Pezzarossi A, Furnari G, Borgia P, Guasticchi G, Giorgi Rossi P. Methods to increase participation in organised screening programs: a systematic review. *BMC Public Health* 2013;**13**: 464.
